# Risk-based regionalization approach for area-wide management of HLB vectors in the Mediterranean Basin

**DOI:** 10.3389/fpls.2023.1256935

**Published:** 2023-12-04

**Authors:** Anaïs Galvañ, Renato Beozzo Bassanezi, Weiqi Luo, Pilar Vanaclocha, Antonio Vicent, Elena Lázaro

**Affiliations:** ^1^Centre de Protecció Vegetal i Biotecnologia, Institut Valencià d’Investigacions Agràries (IVIA), Moncada, Spain; ^2^Departamento de Pesquisa e Desenvolvimento, Fundo de Defesa da Citricultura, Araraquara, SP, Brazil; ^3^Agricultural Research Service, U.S. Department of Agriculture, Fort Pierce, FL, United States; ^4^Center for Integrated Pest Management, North Carolina State University, Raleigh, NC, United States

**Keywords:** Huanglongbing, Spain, area-wide pest management, *Diaphorina citri*, *Trioza erytreae*, regionalization, ClustGeo

## Abstract

Huanglongbing (HLB) is one of the most devastating citrus diseases worldwide. It is associated with the non-culture bacteria *Candidatus* Liberibacter spp., which can be transmitted by grafting and/or the psyllid vectors *Diaphorina citri* (ACP) and *Trioza erytreae* (AfCP). Although HLB has not been reported in the Mediterranean Basin to date, both vectors are present, and thus represent a serious threat to the citrus industry in this region. Resistant citrus cultivars or effective therapeutic treatments are not currently available for HLB. Nevertheless, area-wide pest management via coordinated management efforts over large areas has been implemented in Brazil, China and the USA for HLB control. This study proposes an open access flexible methodology to address area-wide management of both HLB vectors in the Mediterranean Basin. Based on a risk-based approach which considers climatic information and other variables that may influence vector introduction and spread, such as conventional, organic, abandoned and residential citrus areas as well as transportation corridors, an area-wide management division in pest management areas (PMAs) is proposed. The size and location of these PMAs were estimated by means of a hierarchical clustering algorithm with spatial constraints whose performance was assessed under different configuration scenarios. This proposal may assist policymakers and the citrus industry of the citrus-growing areas of the Mediterranean Basin in risk management planning in the case of the spread of HLB vectors or a possible introduction of the disease. Additionally, it may be a valuable resource to inform opinion dynamic models, enabling the identification of pivotal factors for the success of control measures.

## 1 Introduction

Citrus huanglongbing (HLB), or citrus greening, is considered the most devastating bacterial disease of citrus worldwide (Bové, 2006; McCollum and Baldwin, 2016; Jeger et al., 2023). The disease affects citrus trees, causing an overall decline which eventually leads to their death (Hendrichs et al., 2021). HLB is mainly associated with the phloem-limited bacteria *Candidatus* Liberibacter asiaticus (*C*Las), *Ca.* L. africanus (*C*Laf) and *Ca.* L. americanus (*C*Lam). The *C*Las species is the most aggressive form of HLB and is widespread in Asia, North America, South America and Africa (Bové, 2006; Gasparoto et al., 2022). *C*Laf is only present in Africa, while *C*Lam, initially identified in Brazil, is diminishing and being taken over by *C*Las (Teixeira et al., 2005; Gasparoto et al., 2012; Wang, 2020). HLB-associated *Ca.* Liberibacter spp. (*C*Ls) can be transmitted by graft propagation (Bové, 2006), but their natural spread is primarily mediated by two psyllid vectors: the Asian citrus psyllid (ACP), *Diaphorina citri* Kuwayama (Hemiptera: Psyllidae), and the African citrus psyllid (AfCP), *Trioza erytreae* (Del Guercio, 1918) (Hemiptera: Triozidae), native to the Asian and African continents, respectively (Teixeira et al., 2005; Bové, 2006; da Graça et al., 2016; Alquézar et al., 2022).

The pathosystem of HLB involves the pathogen, the plant host and the psyllid vector, interacting in an environmental background that affects the biology of each component and its interactions (Stelinski, 2019). The three species of the genus *Ca.* Liberibacter associated with HLB have a persistent propagative relationship with these psyllid vectors (Carmo-Sousa et al., 2020). As these bacterial pathogens are confined to the phloem, acquisition happens when these psyllid vectors feed on the phloem sap of infected citrus plants, with inoculation occurring via salivation after invading the salivary glands. After acquisition, depending on the psyllid species, transmission can occur within 7-12 days (Canale et al., 2017). Infected adult and/or nymph vectors transmit the bacterium when feeding on healthy flushes, while emerging nymphs acquire it when feeding of them (da Graça et al., 2016; Shimwela et al., 2016). Once the psyllid vector becomes inoculative (able to inoculate plants), it retains this ability for weeks or its whole life, characterizing the transmission as persistent. The HLB-associated bacteria then develop in the tree phloem vascular system, and symptoms take at least four months to appear depending on the tree’s age (Coletta-Filho et al., 2010). The unpredictable lag period between the acquisition of the pathogen and transmission by the psyllid vectors, and for the development of visual symptoms, make HLB eradication by the visual detection of HLB-symptomatic trees problematic.

The *C*Las-ACP combination is widespread in Asia and America whereas *C*Laf-AfCP is the most prevalent in Africa (Alquézar et al., 2022). *C*Las is more heat tolerant than *C*Laf and can develop under relatively low humidity and high temperature conditions. Similarly, ACP is able to adapt to these same climatic conditions. Transmission of *C*Las is closely linked to insect development, where acquisition must occur with a higher efficiency during the nymphal stage (Inoue et al., 2009; Coletta-Filho et al., 2014). Thus, temperature ranges influence transmission rate and disease spread. For instance, in the USA, lower temperatures in California have been suggested to slow down the life cycle of ACP resulting in lower infection rates, whereas the higher temperature range in Florida is more favorable for ACP mating and continued disease progression (McRoberts et al., 2021; Igwe et al., 2022; Hosseinzadeh and Heck, 2023). Conversely, both *C*Laf and AfCP are sensitive to hot and dry conditions, and are thus restricted to areas with relatively cool and humid environments (Catling, 1969; Liu and Tsai, 2000; Bové, 2006; Dala-Paula et al., 2019). Both ACP and AfCP can transmit *C*Las and *C*Laf under experimental conditions (Reynaud et al., 2022), but more research is needed to better understand the epidemiological implications of the *C*Las/AfCP and *C*Laf/ACP combinations.

Nowadays, HLB is widespread in some of the major citrus producing areas worldwide, such as Argentina, Brazil, China, India, Mexico, South Africa and the USA, among others. No therapeutic treatments or citrus cultivars resistant to HLB are currently available. A three-pronged strategy (TPS), consisting of (i) planting of certified healthy citrus material; (ii) removal of inoculum sources-infected trees; and (iii) application of insecticide treatments to control psyllid populations, has been proposed to be key to managing citrus HLB (Bassanezi and Gottwald, 2009; Bové, 2012). Experiences in both Brazil and the USA have highlighted the futility to control HLB without removing infected trees (Alquézar et al., 2022). Consequently, the task of controlling psyllid vectors goes beyond managing current HLB outbreaks; it encompasses the critical mission of preventing future infections. By diminishing the population of infected trees and thereby reducing the reservoir of the bacterium, vector control becomes an essential part of the long-term management of this disease. In essence, vector control is a cornerstone of HLB management because it directly addresses the mode of disease transmission.

Nevertheless, effective control of vector populations can only be achieved through area-wide pest management (Gottwald et al., 2007; Bassanezi et al., 2013). This strategy consists in implementing pest management over extensive areas as a group rather than on individual orchards (Klassen, 2008). It therefore needs the coordination and teamwork of the whole industry including growers, advisers and governmental authorities, among others. This type of management is particularly effective for migratory pest species such as some insect vectors (Dalal et al., 2017; Drmić et al., 2017; Singerman et al., 2017; García-Figuera et al., 2022; International Atomic Energy Agency (IAEA), 2022). Successful cases of area-wide pest management consist of time-coordinated pesticide sprays or other means of control applied simultaneously in an extensive area (Bassanezi et al., 2013; Navarro-Llopis et al., 2014; García-Figuera et al., 2021b; International Atomic Energy Agency (IAEA), 2022).

Area-wide pest management has been attempted for *C*Las/ACP in Brazil, China, Mexico, Argentina, California, Florida and Texas with different aims (Bassanezi et al., 2013; McCollum and Baldwin, 2016; Singerman et al., 2017; McRoberts et al., 2019; García-Figuera et al., 2021b; Hendrichs et al., 2021; Yuan et al., 2021; Alquézar et al., 2022; García-Figuera et al., 2022). In areas where both the vector and the disease are widespread, area-wide management aims to reduce disease intensity and sustain fruit production by combining vector management with the elimination of affected trees and replanting with healthy ones from certified nurseries (Bassanezi et al., 2013; Singerman et al., 2017; García-Figuera et al., 2021b; Alquézar et al., 2022). However, in areas where the vector is widespread but the disease is still geographically restricted, the aim of area-wide management is to limit the spread of the disease to neighboring areas (Grafton-Cardwell and García-Figuera, 2018; García-Figuera et al., 2021b; Yuan et al., 2021; Alquézar et al., 2022).

Citrus-growing areas in Australia, New Zealand and the Mediterranean Basin countries are currently free of HLB (da Graça et al., 2016; McCollum and Baldwin, 2016; European and Mediterranean Plant Protection Organization (EPPO), 2022a; European and Mediterranean Plant Protection Organization (EPPO), 2022b; European and Mediterranean Plant Protection Organization (EPPO), 2022c; García-Figuera et al., 2022). However, in the European Union (EU), *T. erytreae* is currently spreading in the Iberian Peninsula and getting closer to the commercial citrus-growing areas of Spain and Portugal (Arenas-Arenas et al., 2019; Benhadi-Marín et al., 2020), and *D. citri* has recently been reported in Israel (European and Mediterranean Plant Protection Organization (EPPO), 2022) and Cyprus (European and Mediterranean Plant Protection Organization (EPPO), 2023). As indicated by Bové (2006), the introduction of the vector usually precedes subsequent outbreaks of HLB. In fact, during recent years the time lag between vector introduction and HLB outbreaks has been progressively reduced (Alquézar et al., 2022).

The HLB-associated *C*Ls and their psyllid vectors ACP and AfCP have quarantine status in most countries of the Mediterranean Basin, including the EU, where *C*Ls causing HLB are also included in the list of priority pests (European Union (EU), 2019a; European Union (EU), 2019b; Food and Agriculture Organization of the United Nations (FAO), 2022). These EU priority pests are the ones with the highest potential socioeconomic impact. They are subjected to additional mandatory phytosanitary measures including annual surveys, contingency and action plans, simulation exercises, and public information (European Union (EU), 2016; Aragón et al., 2022). Despite the phytosanitary regulations, over the last few years several interceptions of ACP at the EU borders have been reported. Furthermore, irregular imports of propagating plant material constitute another high-risk entry pathway (EFSA PLH Panel et al., 2021). This fact, together with the presence of AfCP in the Iberian Peninsula and ACP in Israel and Cyprus, represents a serious threat to the citrus-growing areas in the Mediterranean Basin (Gutierrez and Ponti, 2013).

The Mediterranean Basin accounts for around 12% of the world’s citrus-growing areas (McCollum and Baldwin, 2016; Siverio et al., 2017; Food and Agriculture Organization of the United Nations (FAO), 2020). Citrus in the Mediterranean are cultivated mainly in coastal areas, forming a virtually continuous belt along the basin. Spain has the largest citrus-growing area in the Mediterranean Basin, with ∼23% of the total surface area (Food and Agriculture Organization of the United Nations (FAO), 2020). In Spain, commercial citrus crops cover nearly 300,000 ha with a production of ∼6.6 million tonnes, making it the main producer in the EU and the sixth in the world (Siverio et al., 2017; Food an Agriculture Organization of the United Nations (FAO), 2020). With a 25% share, Spain is the world’s leading exporter of fresh citrus, with nearly 60% of the production destined to foreign markets. The main citrus region in Spain is the Valencian Autonomous Community, with 54% of the total Spanish citrus-growing area, followed by Andalusia (28%) and the Region of Murcia (13%) (Ministerio de Agricultura, Pesca y Alimentación (MAPA), 2019; Food and Agriculture Organization of the United Nations (FAO), 2020; Ministerio de Agricultura, Pesca y Alimentación (MAPA), 2022). Furthermore, citrus in Spain also has an important sociocultural component and ornamental citrus are widely planted in residential areas (Duarte et al., 2016; Arenas-Arenas et al., 2018; Torregrosa et al., 2019).

The Spanish National Plant Protection Organization (NPPO) developed contingency plans for HLB and its vectors (Ministerio de Agricultura, Pesca y Alimentación (MAPA), 2021a; Ministerio de Agricultura, Pesca y Alimentación (MAPA), 2021b; Ministerio de Agricultura, Pesca y Alimentación (MAPA), 2023a). These contingency plans were recently evaluated in a simulation exercise (Aragón et al., 2022) and have been translated to a specific regulation to address the control and eradication of the AfCP and to prevent the introduction of the ACP and *C*Ls (Ministerio de Agricultura, Pesca y Alimentación (MAPA), 2023b). Nevertheless, how to organize citrus growers in order to coordinate vector control through area-wide management is not specifically addressed in this contingency plan. The particularities of the citrus industry in the Mediterranean Basin, with small orchards (*<*1 ha on average in Spain) often managed independently by individual growers (Reig-Martínez and Picazo-Tadeo, 2004; Fernández-Zamudio et al., 2005; Morell-Monzó et al., 2021), increases their exposure to primary infections, namely those coming from neighboring areas (Bassanezi et al., 2013; Alquézar et al., 2022). The different characteristics of the citrus industry in the Mediterranean Basin limit the extrapolation of the area-wide management programs designed for other citrus-growing areas, thereby making the implementation of area-wide management a challenge. Moreover, area-wide management has not been attempted for AfCP control elsewhere, despite being the vector currently spreading in the Iberian Peninsula.

Considering that ACP and AfCP are now present in the Mediterranean Basin and to prepare for its potential spread to the main citrus-growing areas in the region, the objectives of this study are:

- To define an area-wide management framework for ACP and AfCP in the citrus-growing areas in the Mediterranean Basin. To this end, data from the Valencian Autonomous Community, Spain, and a series of risks potentially associated with the establishment and spread of ACP/AfCP were considered (Flores-Sánchez et al., 2017; McRoberts et al., 2019).
- To optimize the size and location of area-wide management areas. For this purpose, the methodology developed by Gottwald et al (2014a; 2014b). was tailored by adapting a regionalization algorithm and assessing its performance under different configurations of risk homogeneity and spatial aggregation. The methods were implemented in R language (R Core Team, 2021) and, following open science principles, all the underlying code and data necessary to run the tool are made available. This will allow the approach to be updated as new information becomes available, so that end users can adapt it to different epidemiological settings.

In addition, this proposal could provide valuable support to policy makers and the citrus industry in planning for risk management in the event of the spread of HLB vectors or the potential introduction of the disease into citrus-growing regions in the Mediterranean Basin. It can also be used by modelers to improve surveillance and management programs. By integrating epidemiological models with opinion dynamics models that incorporate the principles of area-wide management, it will be possible to determine how best to maximize the potential effectiveness of voluntary HLB control campaigns (Gottwald et al., 2014a; Milne et al., 2020).

## 2 Materials and methods

The Valencian Autonomous Community (ES52 NUTS level 2 EU territorial unit for statistics) was selected as the study area. This region consists of three provinces: Alicante (ES521 NUTS 3), Castellón (ES522 NUTS 3) and Valencia (ES523 NUTS 3). The study area is the region with the largest extension dedicated to citrus in Spain, with characteristics similar to those of other citrus-growing areas in the Mediterranean Basin. Of the three provinces, Valencia has the biggest area devoted to citrus.

The resulting area-wide management zones in our study were defined as Pest Management Areas (PMAs). To facilitate regionalization of the PMAs, the study area was gridded into 24,048 cells of 1 km^2^ using the European Environment Agency (EEA) reference grid downloaded from its official website. This grid is in the ETRS89 Lambert Azimuthal Equal Area (LAEA) projection coordinate reference system (ETRS89/ETRS-LAEA (EPSG:3035)) with a coding system identifying each grid cell with a unique ID (Peifer, 2011).

The original 24,048-cell grid was filtered to identify cells with commercial citrus coverage using a georeferenced dataset from the Spanish Agricultural Plots Geographic Information System (SIGPAC). The SIGPAC dataset provided comprehensive information on the size and shape of commercial citrus orchards and was intersected with the reference grid of the study area. Finally, 7,021 of the original 24,048 cells were identified as cells with commercial citrus coverage. Thus, the grid of the study area was defined by 7,021 cells of 1 km2.

Based on previous studies in other citrus areas (Díaz-Padilla et al., 2014; Gottwald et al., 2014a; Gottwald et al., 2014b), the study area was characterized through different risk factors than can affect the introduction and spread of HLB vectors and, consequently, the disease epidemiology (see **Supplementary Material A** for further details). For this purpose, different georeferenced datasets were exploited to extract data on climatic variables, commercial citrus orchards, population census data and the network of the main transportation corridors. The extracted data were transformed to the ETRS89/ETRS-LAEA (EPSG:3035) reference system using R software version 3.6.0, https://www.R-project.org and aggregated at the cell level into the grid of the study area.

### 2.1 Climatic variables

Hourly data at 2 m for air temperature (K) and dew point temperature (K) at a spatial resolution of 0.1° x 0.1° (∼ 9 km x 9 km) were retrieved from ERA5-Land dataset from 2009 to 2018 in raster format through the Climate Data User (CDS) user interface.

As in Galvañ et al. (2022), relative humidity (RH) as a percentage was calculated according to Wallace and Hobbs (2006) using air and dew point temperatures as follows:


(1) $$RH\;=\;100\cdot \left(\frac{e_{s}\left(Td\right)}{e_{s}\left(T\right)}\right)$$

with *e_s_* (*Td*) and *e_s_* (*T*) denoting actual and saturation vapor pressures in hPa, respectively; with *Td* as the dew point temperature and *T* as the air temperature both in °C and for *e_s_* (*Td*) ≤ *e_s_* (*T*). Actual and saturation vapor pressure were estimated following Bolton (1980) as:


(2) $$e_{s}\left(Td\right)\;=\;6.12\cdot \exp\;\left[(17.67\cdot Td/\left(243.5+Td\right)\right]$$

(3) $$e_{s}\left(T\right)\;=\;6.12\cdot \exp\;\left[(17.67\cdot T/\left(243.5+T\right)\right]$$

Daily maximum, minimum, mean temperature and minimum relative humidity were computed from the hourly variables using the function ‘apply. daily()’ from the R package rts (Babak, 2023) and were extracted to the 1 km^2^ grid of the study area by means of the function ‘extract()’ from the R package raster (Hijmans, 2022). A total of 326 cells were excluded because ERA5-Land does not provide climatic data for land areas adjacent to the coast line (Pelosi et al., 2020) and so the final grid of the study area was defined by 6,695 cells.

### 2.2 Commercial, abandoned and organic citrus

Georeferenced data of organic and abandoned orchards were not available. Thus, the total surface area of organic and abandoned citrus orchards in the study area was obtained from official statistics (Ministerio de Agricultura, Pesca y Alimentación (MAPA), 2019; Ministerio de Agricultura, Pesca y Alimentación (MAPA), 2020). Based on these data, a total of 42.68 km^2^ of citrus under organic production and 103.81 km^2^ of abandoned citrus were randomly assigned to the commercial citrus orchards extracted from the SIGPAC and were first used to filter the grid of the study area. This was done using the function ‘sample()’ from the R package base (R Core Team, 2021). The number of organic/abandoned orchards was determined by dividing the total number of hectares of organic/abandoned by the average commercial orchard size. Consequently, each individual orchard was categorized as in-production, distinguishing between conventional vs. organic management, or abandoned, that is, unmanaged.

In order to characterize each of the cells comprising the study area’s grid in terms of the total surface area under commercial production, including conventional, organic, and unmanaged citrus areas, the the ‘st_intersection()’ function from the R package sf was employed (Pebesma et al., 2023). Through this function, the study area grid was intersected with the georeferenced orchard database, and the total area for each type of orchard was computed by aggregating the surfaces of each orchard intersected by a cell.

### 2.3 Residential citrus

Two sources of data were exploited for residential citrus and subsequently combined: i) daily minimum temperatures, and ii) population census data (see Section 2.1 for further details).

To account for the detrimental effect of cold conditions in citrus growth, daily minimum temperatures in January were averaged for each year from 2009 to 2018. Based on Davies and Albrigo (1999) and Bijzet (2006), a temperature threshold of 2.5°C was chosen to draw an isotherm defining those parts of the study area in which the growth of citrus is not limited by cold temperatures *T* ≥ 2.5°C.

Population census data at country level (ES NUTS 1) were extracted from the GEOSTAT population grid, which provides data on population density as the number of inhabitants per 1 km^2^ cell at the EEA reference grid (EUROSTAT, 2016). The ratio between the residential citrus coverage and the number of inhabitants was estimated based on the census of citrus trees for the city of Seville (Cabanillas, 2020), in Andalusia, and its population (Instituto Nacional de Estadística (INE), 2020). This city was chosen for having the most complete and updated census on residential citrus trees in Spain. The number of residential citrus trees in Seville was transformed to residential citrus area, assuming a coverage area of 20 m^2^ per tree. The estimated residential citrus area was divided by the total number of inhabitants, resulting in a ratio of 1.33·10^−6^ km^2^ of residential citrus area per inhabitant.

This ratio was applied to the number of inhabitants in each 1 km^2^ cell, but considering only those cells defined by the isotherm as suitable for citrus growth. Finally, only those cells classified as “suitable” within the grid of the study area were taken into account. As a result, the information of the residential citrus area was integrated into the study area cells, along with the information of the total citrus area in conventional and organic production and the “abandoned” citrus area.

### 2.4 Transportation corridors

The road transport network at country level (ES NUTS 1) was downloaded from the National Geographic Information Centre (CNIG) of Spain (Centro Nacional de Información Geográfica (CNIG), 2021). The main transportation corridors were extracted from this dataset choosing only motorways and highways in the study area, as they are considered the primary routes for the transport of citrus fruit and planting materials.

### 2.5 Pest management areas (PMAs)

#### 2.5.1 Risk factors

**CLIMATIC SUITABILITY**. The risk factor associated with climatic suitability was calculated separately for ACP and AfCP. As with most insects, the psyllid vectors of HLB are strongly influenced by climatic conditions, particularly temperature and relative humidity. Two risk components related to the potential number of generations (*R_png_*) and the number of favorable days for development (*R_nfd_*) were considered for both vectors (see **Supplementary Material A** for further details). To estimate these two risk components, daily data of temperature and relative humidity for the period 2009-2018 were used (see Section 2.1).

The potential number of generations per year *R_png,i_* for each cell *i* was calculated based on the average cumulative degree-days for the 10-year period (2009-2018) and dividing by the degree-days (*DD*) required for an egg of ACP/AfCP to become an adult above a developmental threshold temperature (*T_l_*) as follows:


(4) $$R_{png,i}\;=\;\frac{\frac{\left[\left(\sum^{\;}T_{mean_{day_{j},year_{k}},i}\right)-\left(T_{l}\cdot n_{days}\right)\right]}{n_{years}}}{DD}$$

where $T_{mean_{day_{j},year_{k}},i}$ denotes the daily mean temperature for day *j* of year *k* within cell *i* with *j* = 1,2*,…*,365, *k* = 1,2*,…*,10 and $n_{days\left(years\right)}$ denoting the total number of days and years considered, respectively.

For ACP, *T_l_* = 10.45°C and *DD* = 249.88°C were the values selected according to Liu and Tsai (2000). For AfCP, *T_l_* = 10°C and *DD* = 270.00°C based on the work of Catling (1973) and Aidoo et al. (2022).

The number of favorable days *R_nfd,i_* for each cell *i* was calculated considering only those days allowing the development of all the stages of the insect. For ACP, “a favorable day” (*FD_acp_*) was defined considering that this insect species is able to develop with mean temperatures (*T_mean_*) between 10°C, as a lower threshold (*T_l_*), and 33°C, as an upper threshold (*T_u_*) (Liu and Tsai, 2000):


(5) $$FD_{acp}=\{\begin{array}{ccc} & 1 & \text{if }T_{mean}\;>\;10^{\circ }\text{C and }T_{mean}\;<\;33^{\circ }\text{C}\\ & 0 & ,\text{otherwise}. \end{array}$$

According to Aidoo et al. (2022), a “favorable day” for AfCP (*FD_afcp_*) was defined considering that it is able to develop at mean temperatures (*T_mean_*) between 10°C, as *T_l_*, and 27°C, as *T_u_* and also including a saturation deficit (*SD*) ≤ 32.1 mmHg (Catling, 1969; Catling, 1972). This insect species is particularly sensitive to the effects of high temperature and low relative humidity, which are combined in the *SD* index.


(6) $$FD_{afcp}=\{\begin{array}{ccc} & 1 & \text{if }T_{mean}\;<\;10^{\circ }\text{C and }T_{mean}\;>\;27^{\circ }\text{C and }SD\;\leq \;32.1\text{ mmHg}\\ & 0 & ,\text{otherwise}. \end{array}$$

*SD* in hPa was estimated following Murray (1967) as,


(7) $$SD\;=\;\left(\frac{(100-RH_{min)}}{100}\right)\cdot e_{s}\left(T_{max}\right)$$

where *RH_min_* and saturation vapor pressure (*e_s_*) were estimated from hourly *RH* following equations 1 and 2, respectively, but considering maximum daily temperature (*T_max_*). To convert *SD* from hPa to mmHg, the estimates were multiplied by 0.7501.

Thus, the *R_n f d,i_* for each cell *i* was computed as:


(8) $$R_{nfd,i}\;=\;\frac{\sum^{\;}FD_{day_{j},year_{k}}}{n_{year}},$$

where $FD_{day_{j},year_{k}}$ is an indicator variable of a “favorable day” for day *j* of year *k* within cell *i* with *j* = 1,2*,…*,365, *k* = 1,2*,…*,10 and *n_year _* denoting the total number of years.

The psyllids ACP and AfCP lay eggs on leaf flushes and their immature stages feed on tender foliar tissues but not on mature leaves (Annecke and Cilliers, 1963; Moran and Buchan, 1975; van den Berg and Deacon, 1988; Hall, 2020; Monzó and Vanaclocha, 2023). Therefore, daily climate variables were filtered considering only the days from 15 February to 30 April, June and October, assuming that under Mediterranean conditions these are the three major leaf flushing periods (Garcia-Marí et al., 2002) (See **Supplementary Material A** for further details).

**Figures SA1**, **SA2** (**Supplementary Material A**) describe the potential number of generations and the favorable days estimated for the study area.

**COMMERCIAL CITRUS AREA**. The risk factor associated with the commercial citrus area for each cell *i*, *R_com,i_*, was calculated as:


(9) $$R_{com,i}\;=\;CCA_{i},$$

where *CCA_i_* denotes the commercial citrus area in km^2^ for each cell *i*.

**ABANDONED/ORGANIC ORCHARDS.** The risk factors associated with abandoned and organic orchards for each cell *i*, *R_abn_*_(_*_org_*_)_*_,i_*, were computed considering the presence of abandoned or organic orchards within a cell *i* and/or in its *j* neighboring cells. The baseline risk associated for each cell *i*, *R*0*_abn_*_(_*_org_*_)_*_,i_*, was computed as:


(10) $$R0_{abn\left(org\right),i}=\{\begin{array}{ccc} & CCA_{i} & \text{if }A\left(O\right)CA_{i}\;=\;0\\ & CCA_{i}\;\cdot F_{abn\left(org\right)} & \text{if }A\left(O\right)CA_{i}\;\neq \;0, \end{array}$$

where *CCA_i_* and *A*(*O*)*CA_i_* denote the commercial and abandoned/organic citrus area in km^2^ for each cell *i*, respectively; *F_abn_*_(_*_org_*_)_ is an amplification coefficient that increases the risk in those cells *i* in which abandoned/organic citrus orchards are present. The values for this coefficient were set based on the study by Martini et al. (2016). In this work, the overwintering abundance of adult ACP was evaluated in commercial citrus orchards under conventional or organic management as well as those that have been abandoned. The amplification coefficient was then calculated based on the average abundance of adults in organic (3) and abandoned orchards (19) relative to that in commercial orchards (0.50) as *F_abn_* = 38 = 19*/*0.50 and *F_org_* = 6 = 3*/*0.50. In the absence of this type of information for AfCP, the same values of ACP were used.

The *R_abn_*_(_*_org_*_)_*_,i_* for each cell *i* was calculated as:


(11) $$R_{abn\left(org\right),i}\;=\;R0_{abn\left(org\right),i}+\sum_{j\;=\;1}^{max=8}R0_{abn\left(org\right),j},$$

where cell *i* and each cell *j* were considered neighbors if the distance between their centroids did not exceed 1,500 m based on van den Berg and Deacon (1988) and Lewis-Rosenblum et al. (2015) (see **Supplementary Material A** for further details). Thus, the maximum number of neighbor cells *j* per cell *i* was 8.

**RESIDENTIAL CITRUS**. The risk factor associated with the proximity of residential citrus (*R_res,i_*) for each cell *i* was calculated following the same approach used for *R_abn_*_(_*_org_*_)_*_,i_*. The presence of residential citrus within a cell *i* and/or the proximity of cells with residential citrus was considered using the same criterion of proximity between cells and, thus, the baseline risk associated *R*0*_res,i_* for each cell was computed as:


(12) $$R0_{res,i}=\{\begin{array}{ccc} & CCA_{i} & \text{ if }RCA_{i}\;=\;0\\ & CCA_{i}\;\cdot F_{res} & \text{ if }RCA_{i}\;\neq \;0 \end{array}$$

where *CCA_i_* and *RCA_i_* denote the area (%) covered by commercial and residential citrus for each cell *i*, respectively. Due to the lack of information, *F_res_* was set as 6, assuming that the presence/proximity of residential citrus would affect the introduction and spread of ACP and AfCP in a similar way to the presence/proximity to organic orchards.

The *R_res,i_* for each cell *i* was calculated as:


(13) $$R_{res,i}\;=\;R0_{res,i}+\sum_{j\;=\;1}^{max=8}R0_{res,j}.$$

**TRANSPORTATION CORRIDORS**. The spread of ACP and AfCP can be influenced by human activities, such as the transportation of infested plants and fruits. Trucks transporting citrus fruit from orchards to packing houses have been identified as a means of HLB-vector spread (Halbert et al., 2010). In our case, the motorways and highways within the study area were considered the main transportation corridors for commercial citrus fruit and planting material. The risk factor of transportation corridors, *R_tra,i_*, was estimated considering the presence of this type of roads within a cell *i* and/or their close proximity. As in the other risk factors, the criterion of proximity between cells was also established considering a maximum distance between centroid cells *d_i,j_* ≤ 1,500 m. The associated baseline risk *R*0*_tra,i_* for each cell was computed as:


(14) $$R0_{tra,i}=\{\begin{array}{ccc} & CCA_{i} & \text{ if }I_{tra,i}\;=\;0\\ & CCA_{i}\cdot F_{tra} & \text{ if }I_{tra,i}\;\neq \;0 \end{array}$$

where *CCA_i_* denotes the area (%) covered by commercial citrus and *I_tra,i_* is an indicator variable that was set as equal to 1, *I_tra,i _* = 1, if a transportation corridor was present in the cell *i*; otherwise, it was set as equal to 0. Due to the lack of information and for the sake of simplicity, the corresponding amplification coefficient was set at 6, *F_tra _* = 6, considering that the presence/proximity of a cell *i* to a transportation corridor would affect the introduction and spread of ACP and AfCP in a similar manner.

The *R_tra,i_* for each cell *i* was calculated as:


(15) $$R_{tra,i}\;=\;R0_{tra,i}+\sum_{j\;=\;1}^{max=8}R0_{tra,j}.$$

**RISK FACTOR NORMALIZATION**. The values of the risk factors were scaled from 0 to 1 using the min-max normalization. For each risk factor, its minimum value was transformed into 0 and its maximum value into 1 and, thus, every other value was transformed into a decimal between 0 and 1 as follows:


(16) $$R_{i}^{*}\;=\;\frac{R_{i}-\text{min}\left(R\right)}{\text{max}\left(R\right)-\text{min}\left(R\right)}.$$

Here, *R_,i_* is the risk value associated with a cell *i*, and min(*R*) and max(*R*) are the minimum and maximum values of the risk factor *R*, respectively, which define the range of this risk factor *R* within the study area.

**OVERALL RISK**. To compute the overall risk, *OR_i_*, for each cell *i*, two approaches were followed. In the absence of information on the relative importance of each risk factor, the first one consisted in averaging them:


(17) $$\begin{array}{l} OR_{1,i}=\frac{1}{m}\sum_{m=1}^{M=7}R_{m,i}^{*}\\ \;=\frac{1}{m}\left(R_{png,i}^{*}+R_{nfd,i}^{*}+R_{com,i}^{*}+R_{abn,i}^{*}+R_{org,i}^{*}+R_{res,i}^{*}+R_{tra,i}^{*}\right). \end{array}$$

When information is available from epidemiological studies or expert consultancy, a second approach may be used, assigning a weight (*w_m_*) to each of the *m*-th normalized individual risks and calculating a weighted average such as follows:


(18) $$\begin{array}{l} OR_{2,i}=\sum_{m=1}^{M=7}R_{m,i}^{*}\cdot w_{m}\text{ with}\sum_{m=1}^{M=7}w_{m}\;=\;1\\ \;=\left(w_{1}\cdot R_{png,i}^{*}+w_{2}\cdot R_{nfd,i}^{*}+w_{3}\cdot R_{com,i}^{*}+w_{4}\cdot R_{abn,i}^{*}+w_{5}\cdot R_{org,i}^{*}+w_{6}\cdot R_{res,i}^{*}+w_{7}\cdot R_{tra,i}^{*}\right), \end{array}$$

with *w*_1_ = *w*_2_ = *w*_3 =_ 0.07, *w*_4 =_ 0.43, *w*_5 =_ 0.21, *w*_6 =_ 0.11 and *w*_7 =_ 0.04. In our case, those values were set considering a higher weight for the risks associated to abandoned and organic orchards. The two above-mentioned approaches were estimated for both ACP and AfCP. Consequently, *OR*_1_ and *OR*_2_ were separately determined for ACP and AfCP, respectively. The variations in these estimates are due to the differences in the biology of these two isnect vectors, which affect the calculation of *R_png_* and *R_nfd_*, as justified above in the section **CLIMATIC SUITABILITY**. The overall risk factors were also scaled from 0 to 1 following min-max normalization as described above.

#### 2.5.2 PMAs size and location: regionalization algorithm

The Ward-like hierarchical clustering algorithm from the R package ClustGeo (Chavent et al., 2021) was used to set the size and location of the PMAs. This algorithm makes it possible to include spatial constraints optimizing the convex combination of two dissimilarity matrices *D*_0_ and *D*_1_ and a mixing parameter *α* ∈ [0,1], *D_α_* = (1 − *α*)*D*_0_ + *αD*_1_. The *D*_0_ matrix captures the dissimilarities in the “feature space” (i.e., estimated from the overall risk) whereas the *D*_1_ gives the dissimilarities in the “constraint space” (i.e., estimated from the distance between observations). The parameter *α* quantifies the relative importance of *D*_0_ as compared to *D*_1_ in the clustering procedure. Specifically, when *α* = 0, the spatial dissimilarities are not taken into account and when *α* = 1, the overall risk distances are not considered and PMAs are estimated considering only the spatial distances (Chavent et al., 2018; Camêlo Aguiar et al., 2020). In this respect, the package ClustGeo also includes a functionality to address the choice of the parameter *α*.

Risk factors were calculated for the Valencian Autonomous Community (ES52 NUTS level 2), but the proposed methodology for PMA delineation was evaluated in the two most representative counties (Intitut Valencià d’ Estadística (IVE), 2022) within the study area in terms of commercial citrus areas. These two counties, “Camp de Túria” and “Ribera Alta”, are located in the province of Valencia (ES523 NUTS 3). A county (i.e., a *comarca* in Spanish) is a territorial division legally defined by each autonomous community in Spain (NUTS 2 regions) that groups together several municipalities [LAUs, (EUROSTAT, 2021)] within NUTS 3 regions with the aim of providing common public services. PMAs were set at county level owing to the smallholding structure of citrus in the study areas as well as the potential role of agricultural cooperatives to coordinate programs for pest control (Fernández-Zamudio et al., 2005; Meliá-Martí, 2021), which operate mainly at county level.

The methodology was evaluated separately for the two overall risks described above and both insect vectors, resulting in 4 scenarios. In particular, PMAs regionalization for ACP and AfCP management was developed on the two overall risks computed (*OR*_1_: *OR*_1_*_,apc _* and *OR*_1_*_,afcp _* and *OR*_2_: *OR*_2_*_,apc _* and *OR*_2_*_,afcp_*). The algorithm was run independently for both counties under the following conditions:

- *D*_0_ matrix was computed from the Euclidean distance between the *n* cells performed for the overall risk variable.
- *D*_1_ matrix was computed from the shortest Euclidean distance in geographical space between the *n* cell centroids.
- The choice of the maximum number *K* of PMAs (i.e., clusters) was defined assuming an optimum PMA size of 25 cells, i.e., by dividing the total number of cells within a county by 25. Those counties with fewer than 25 cells of citrus were not considered in the regionalization process.

The performance of the regionalization method was evaluated by a sensitivity analysis defined on the basis on three values of the mixing parameter: *α* = 0.1, *α* = 0.9 and the optimal value suggested by Clustgeo that balances the loss of risk homogeneity and the gain in spatial aggregation. The choice of *α* = 0.1 underscored the significance of risk distances, whereas *α* = 0.9 highlighted the importance of spatial distances in the regionalization procedure. The exploration of various *α* values was aimed to provide a thorough evaluation of how this parameter influences clustering outcomes. Despite the package ClustGeo includes a functionality to address the choice of the parameter *α*, it was proposed to explore these two extreme values, *α* = 0.1 and *α* = 0.9, against the proposed “optimal value” to indentify the value that makes the most practical sense.

Several internal validation measures were calculated to quantify compactness and/or separation (Leskovec et al., 2014; Kassambara, 2017). Compactness measures how close the objects are within the same PMA, estimated by the *complete diameter* as the distance between the two most remote cells within the same PMA and summarized by its maximum value. Separation measures how well separated a PMA is from the others, estimated by means of the *complete linkage distance* as the distance between the most remote cells belonging to two different PMAs and summarized by its minimum value. The Dunn index (*D*) (Dunn, 1973; Dunn, 1974) was estimated from the *max*(*complete.diameter*) and the *min*(*complete.linkage.distance*) as follows:


(19) $$D=\frac{min\left(complete.linkage.distance\right)}{max\left(complete.diameter\right)}.$$

Thus, those values of *α* resulting in compact and well-separated PMAs will have a higher value of the Dunn index. The above-mentioned internal validation measures were computed using the R package clv (Nieweglowski, 2020). Compactness and separation were also evaluated visually by comparing overall risk distribution between PMAs using boxplots. Additionally, the normalized proportion of pseudo-inertia (Chavent et al., 2018) was computed for each *α* value to quantify the loss of risk homogeneity and spatial aggregation in relation to the reference configurations *α* = 0 (i.e., no spatial information used to define PMAs and risk homogeneity is assumed to be 1 (100%)) and *α* = 1 (i.e., only spatial information used to define PMAs and spatial aggregation is assumed to be 1 (100%)), respectively.

## 3 Results

### 3.1 Description of study area

**Figure 1:**
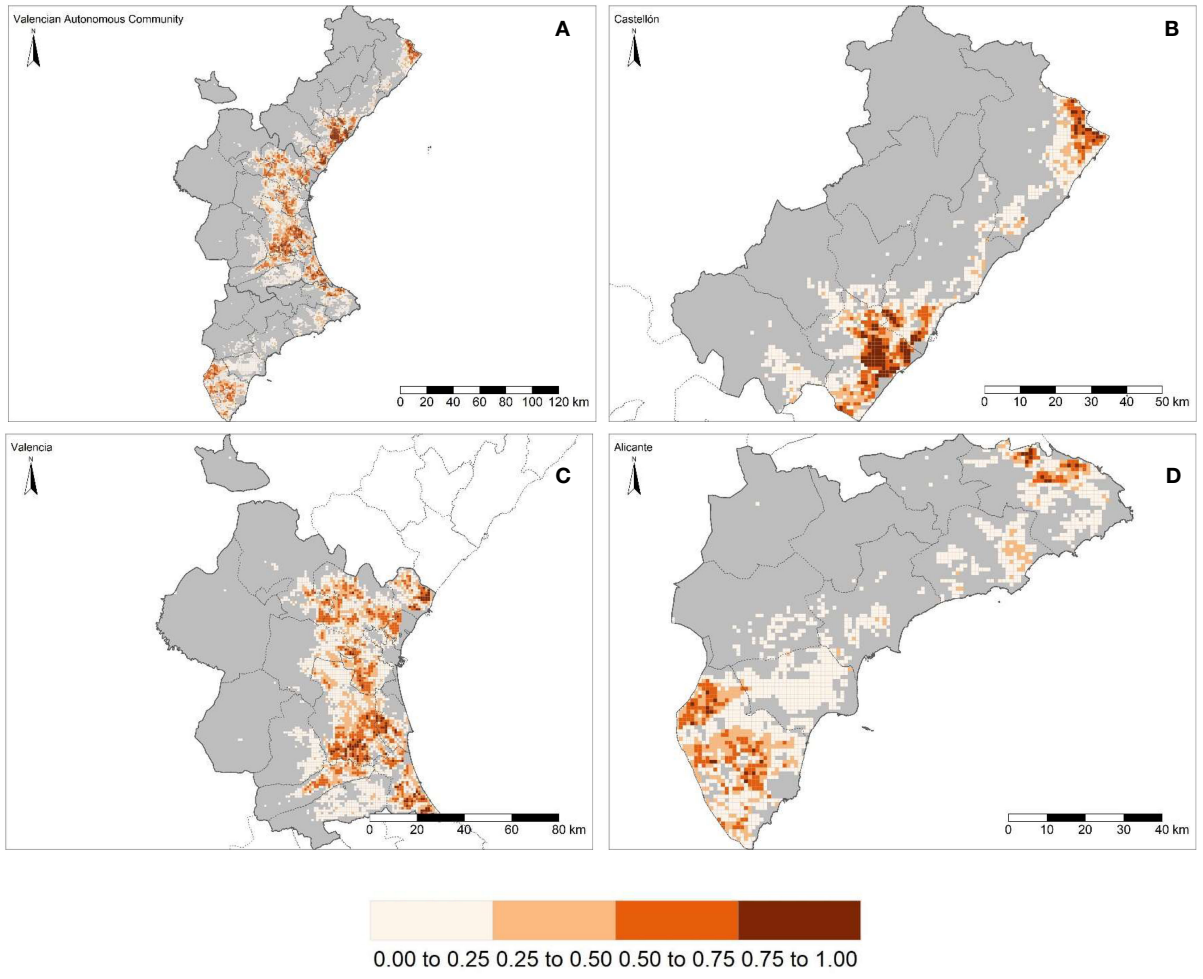
Commercial citrus density per km^2^ within the Valencian Autonomous Community **(A)** (ES52 NUTS 2) and disaggregated at province level (NUTS 3): Castellón **(B)**, Valencia **(C)** and Alicante **(D)**. The continuous red line shows the boundaries of “El Camp de Túria” (top) and “La Ribera Alta” counties (bottom) within the province of Valencia **(C)**. Together, the two counties represent the largest portion of the commercial citrus-growing area.

The study area was mapped onto a grid of 6,695 cells in accordance with the EEA reference grid, of which 3,550 (53.02%) are in the province of Valencia, 1,835 (27.41%) are in the province of Alicante and the remaining 1,310 (19.57%) are located in the province of Castellón. The study area has a total of 1,635.53 km^2^ of commercial citrus, with the largest area in Valencia, with 943.95 km^2^, followed by Castellón and Alicante, with 354.10 and 337.82 km^2^, respectively (**Figure 1**; **Table 1**). “El Camp de Túria” and “La Ribera Alta” counties, both in the province of Valencia, were chosen for the evaluation of the regionalization methodology to delineate the size and location of PMAs (see section 3.3). These two counties (**Figure 1B**) represent 44.54% of the total citrus area (i.e., commercial and residential) in the province and 25.70% of that in the study area.

**Table 1:** Number of EEA reference grid cells, commercial citrus-growing area: conventional, organic and abandoned areas, and residential citrus area in the Valencian Autonomous Community (ES52 NUTS level 2) and disaggregated at province level (NUTS level 3).

	N cells (1 km^2^)	Commercial^*^	Conventional^*^	Organic^*^	Abandoned^*^	Residential^*^
Castellón (ES522 NUTS 3)	1,310 (19.57%)	354.10 (100%)	324.59 (91.67%)	8.62 (2.43%)	20.89 (5.90%)	0.49 (16.43%)
Valencia (ES523 NUTS 3)	3,550 (53.02%)	943.95 (100%)	862.94 (91.42%)	23.47 (2.49%)	57.55 (6.10%)	1.84 (62.49%)
Alicante (ES521 NUTS 3)	1,835 (27.41%)	337.48 (100%)	307.74 (91.19%)	8.62 (2.55%)	21.11 (6.26%)	0.62 (21.08%)
Valencian A.C. (NUTS 2)	6,695 (100%)	1,635.53 (100%)	1,495.27 (91.42%)	40.71 (2.49%)	99.55 (6.09%)	2.95 (100%)

^*^ Area in km^2^

Of the 1,635.53 km^2^ (100%) of commercial citrus-growing area, 1,495.27 km^2^ (91.42%) are under conventional management, 40.71 km^2^ (2.49%) are organic and the remaining 99.56 km^2^ (6.09%) are unmanaged (i.e., abandoned). Of the 354.10 km^2^ (100%) of commercial citrus-growing area in the province of Castellón, 324.59 km^2^ (91.67%), 8.62 km^2^ (2.43%) and 20.98 km^2^ (5.90%) are conventional, organic and abandoned, respectively. The 943.95 km^2^ of commercial citrus-growing area in the province of Valencia are distributed into 862.94 km^2^ (91.42%) under conventional management, 23.47 km^2^ (2.49%) are organic and 57.55 km^2^ (6.10%) are abandoned. Of the 337.48 km^2^ of commercial citrus-growing area in Alicante, 307.74 km^2^ (91.19%) are under conventional management, 8.62 km^2^ (2.55%) are organic and 21.12 km^2^ (6.26%) are abandoned. A total of 2.95 km^2^ of residential citrus were estimated for the whole study area, with 0.49 km^2^ (16.43%) in the province of Castellón, 1.84 km^2^ (62.49%) in the province of Valencia and 0.62 km^2^ (21.08%) in the province of Alicante (**Table 1**).

### 3.2 Overall risk

**Figure 2:**
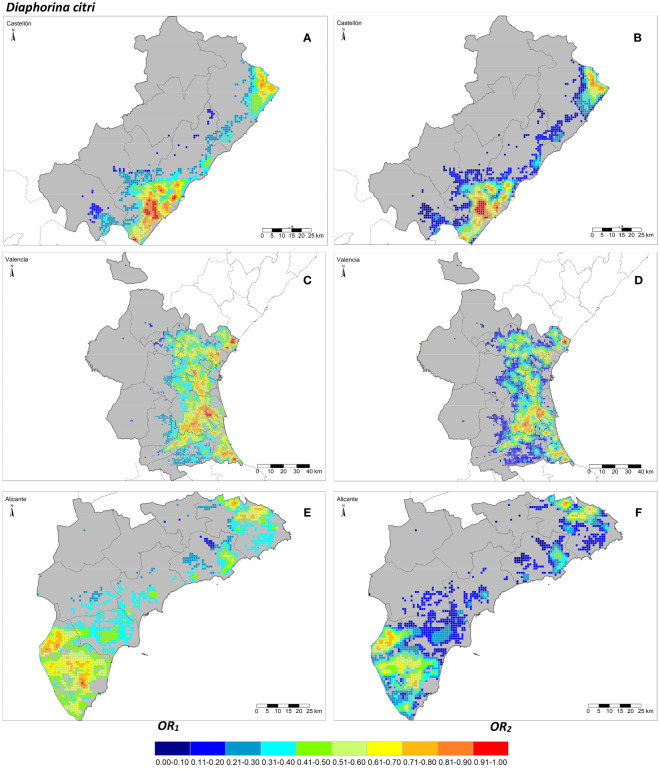
Overall risk 1 (*OR*_1_*_,acp_*) **(A, C, E)** and 2 (*OR*_2_*_,acp_*) **(B, D, F)** for *Diaphorina citri* in the Valencian Autonomous Community disaggregated at province level (NUTS 3): Castellón **(A, B)**, Valencia **(C, D)** and Alicante **(E, F)**.

**Figure 3:**
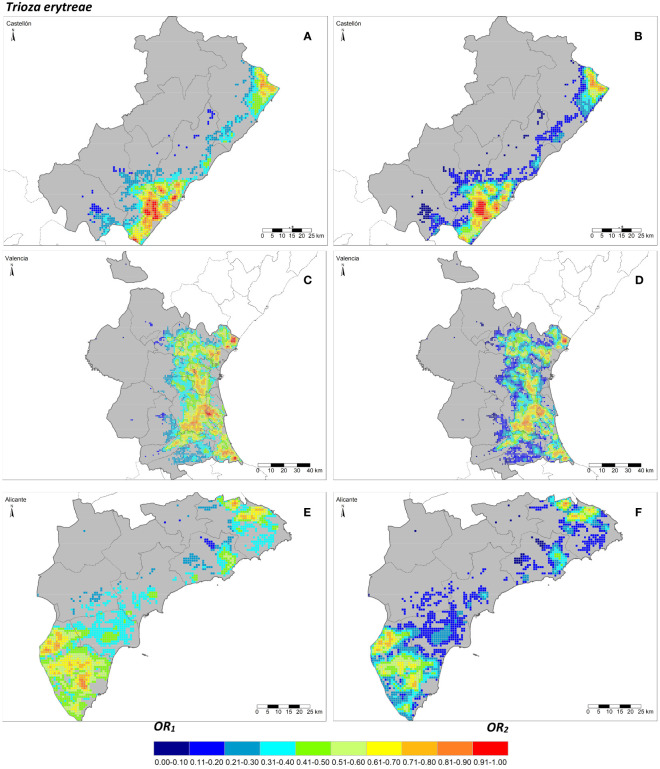
Overall risk 1 (*OR*_1_*_,afcp)_* **(A, C, E)** and 2 (*OR*_2_*_,afcp)_* **(B, D, F)** for *Trioza erytreae* within the Valencian Autonomous Community (ES52 NUTS 2) disaggregated at province level (NUTS 3): Castellón **(A, B)**, Valencia **(C, D)** and Alicante **(E, F)**.

**Figure 4:**
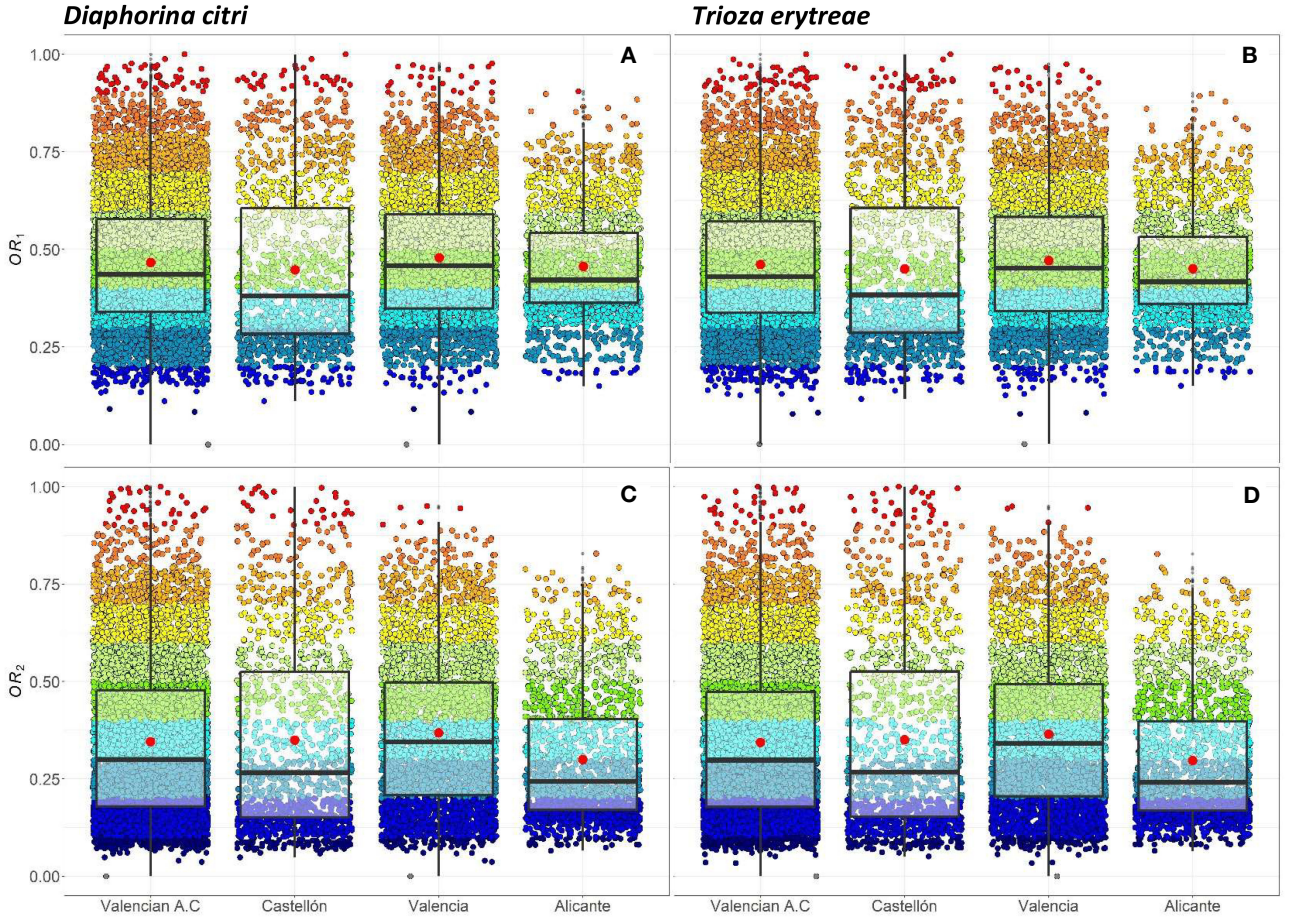
Individual (per cell) and overall distribution of the *OR*_1_*_,acp _* **(A)**, *OR*_1_*_,afcp_* **(B)** and the *OR*_2_*_,acp _* **(C)**, *OR*_2_*_,afcp_* **(D)** within the Valencian Autonomous Community (ES52 NUTS 2) disaggregated at province level (NUTS 3): Castellón, Valencia and Alicante for *Diaphorina citri* (acp) **(A, C)** and *Trioza erytreae* (afcp) **(B, D)**. Box-and-whisker plots for **(A–D)** red dots represent the mean value; central line represents the median value; box limits represent the first (*Q*_1_) and the third (*Q*_3_) quantiles; upper whisker represents *min*(*max*(*x*)*,Q*_3 +_ 1.5*IQR*); lower whisker represents *max*(*min*(*x*)*,Q*_1_ − 1.5*IQR*); *IQR* = *Q*_3_ − *Q*_1_; outliers are represented by dots.

The seven risk factors were summarized by two overall risk factors, *OR*_1_ and *OR*_2_, for ACP and AfCP in the study area (**Figures 1**, **2**). In general, *OR*_2_ presented lower values than *OR*_1_ for both vectors, as observed in **Figures 2B ,D, F** and **3B, D, F**, where a greater predominance of cooler colors is observed in relation to **Figures 2A, C, E** and **3A, C, E**. **Figure 4** describes the range of the four distributions capturing *OR*_1_ and *OR*_2_ for both ACP and AfCP and shows the same trend as that noted above.

The distributions associated with *OR*_1_ (**Figures 4A, B**) have a smaller interquartile range, $IQR=Q_{3}-Q_{1}$ , than those associated with *OR*_2_ (**Figures 4C, D**) for both insect vectors (**Figures 4A, C** for ACP and **Figures 4B, D** for AfCP). Hence, the differences between cells for *OR*_1_ risk are smaller than for *OR*_2_ risk. The median values (*Mdn*.) for *OR*_1_ are higher than those corresponding to *OR*_2_. However, for both overall risks, the differences between the distributions for ACP and AfCP are minor. Specifically, for ACP: $Mdn._{OR_{1,acp}}\;=\;0.44$ and $Mdn._{OR_{2,acp}}\;=\;0.30$ (**Figures 4A, C**), whereas for AfCP: $Mdn._{OR_{1,afcp}}\;=\;0.43$ and $Mdn._{OR_{2,afcp}}\;=\;0.30$ (**Figures 4B, D**). Likewise, the means of the distributions (*M.*) for ACP are $M._{OR_{1,acp}}\;=\;0.47$ and $M._{OR_{2,acp}}\;=\;0.35$ (**Figures 4A, C**), whereas for AfCP $M._{OR_{1,afcp}}\;=\;0.42$ and $M._{OR_{2,afcp}}\;=\;0.33$ (**Figures 4B, D**).

The overall risk factor trends described above for the study region are also reproduced at province level, when comparing between the two overall risk factors, *OR*_1_ vs. *OR*_2_, as well as between the two insect vectors, ACP and AfCP. The province of Alicante showed the smallest differences between cells in terms of overall risk values, with an *IQR* lower than the other two provinces. The province of Valencia, which has the largest percentage of the citrus area and therefore the highest number of cells (**Table 1**), presents a trend similar to that of the whole study area (**Figure 4**).

### 3.3 PMAs

#### 3.3.1 Diaphorina citri

**Figure 5:**
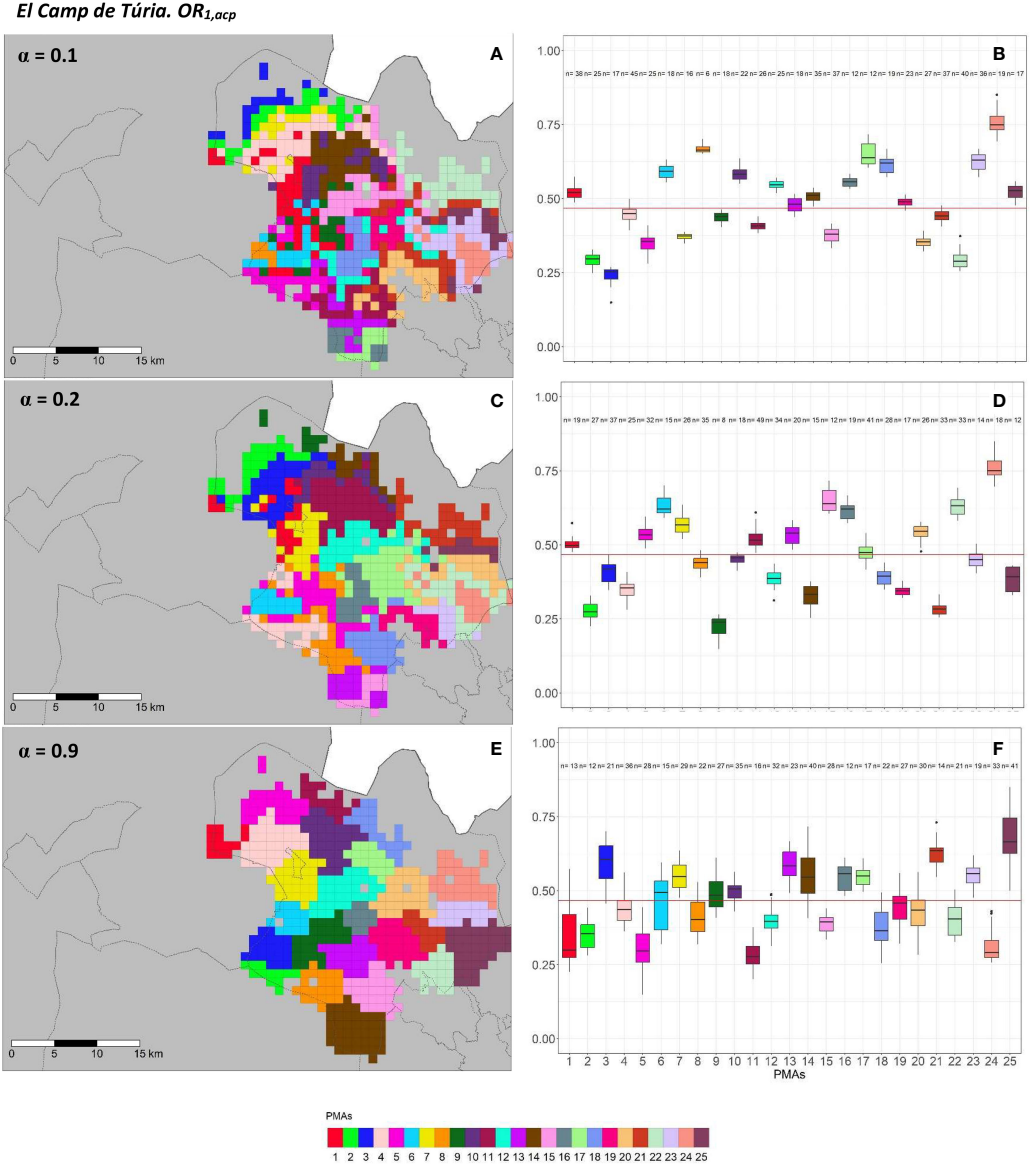
Spatial **(A, C, E)** and overall distribution **(B, D, F)** of the delimited PMAs for *OR*_1_*_,acp _* **(A, C, E)** in “El Camp de Túria” county with *α* of 0.1 **(A, B)**, 0.3 **(C, D)** and 0.9 **(E, F)**. In box-and-whisker plots red line represents the mean value of *OR*_1_*_,acp _* in “El Camp de Túria” county; n quantifies the size of each Pest Management area in terms of the number of 1 km^2^ cells; central line represents the median value; box limits represent the first (*Q*_1_) and third (*Q*_3_) quantiles; upper whisker represents $min\left(max\left(x\right),Q_{3}+1.5IQR\right)$ ; lower whisker represents $max\left(min\left(x\right),Q_{1}-1.5IQR\right)$ ; $IQR=Q_{3}-Q_{1}$ ; outliers are represented by dots.

**Figure 6:**
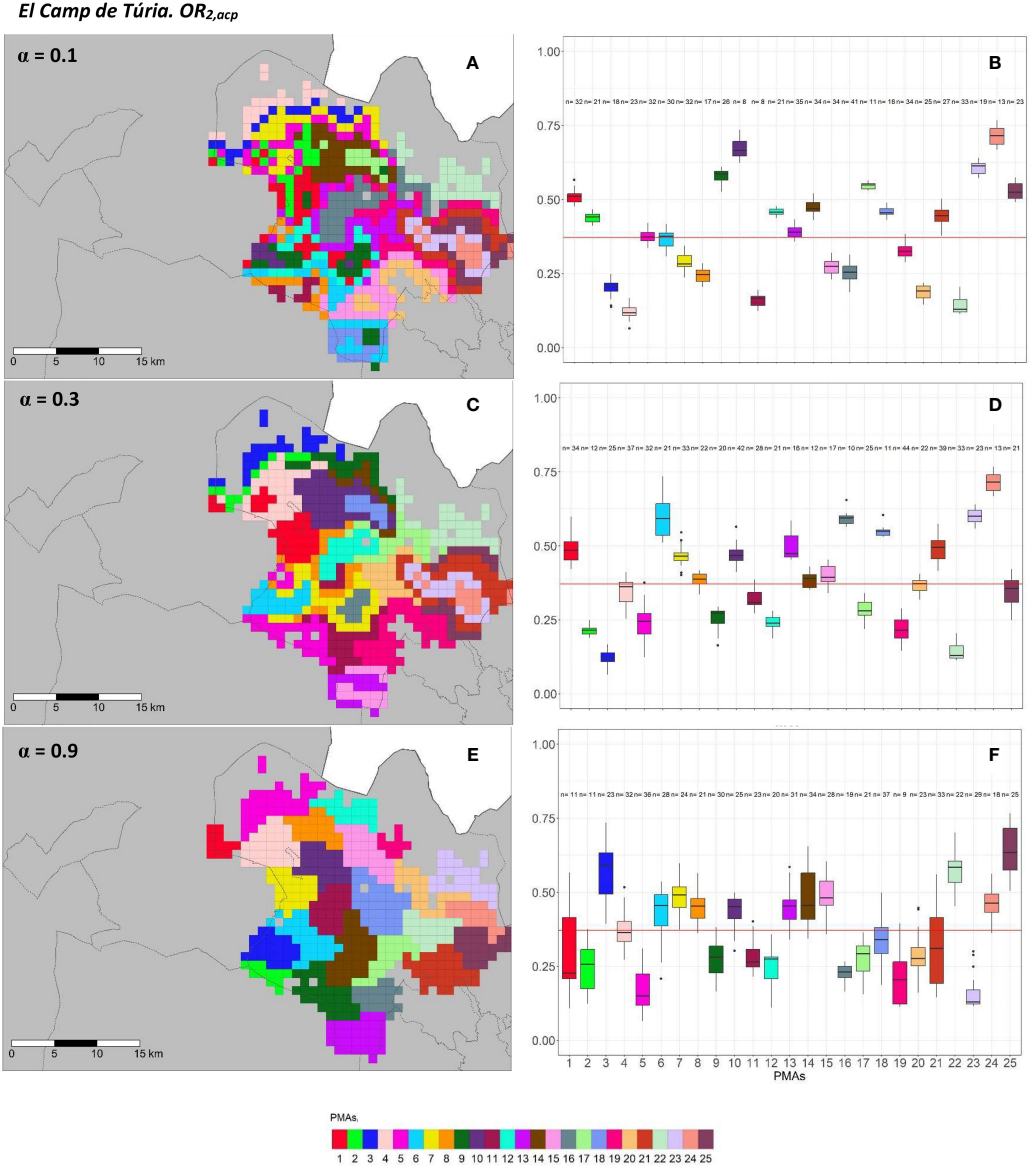
Spatial **(A, C, E)** and overall distribution **(B, D, F)** of the delimited PMAs for *OR*_2_*_,acp_* **(A, C, E)** in “El Camp de Túria” county with *α* of 0.1 **(A, B)**, 0.3 **(C, D)** and 0.9 **(E, F)**. In box-and-whisker plots red line represents the mean value of *OR*_2_*_,acp _* in “El Camp de Túria” county; n quantifies the size of each Pest Management Area in terms of the number of 1 km^2^ cells; central line represents the median value; box limits represent the first (*Q*_1_) and third (*Q*_3_) quantiles; upper whisker represents *min*(*max*(*x*)*,Q*+ 1.5*IQR*); lower whisker represents *max*(*min*(*x*)*,Q^1^*− 1.5*IQR*); $IQR=Q_{3}-Q_{1}$ ; outliers are represented by dots.

**Figure 7:**
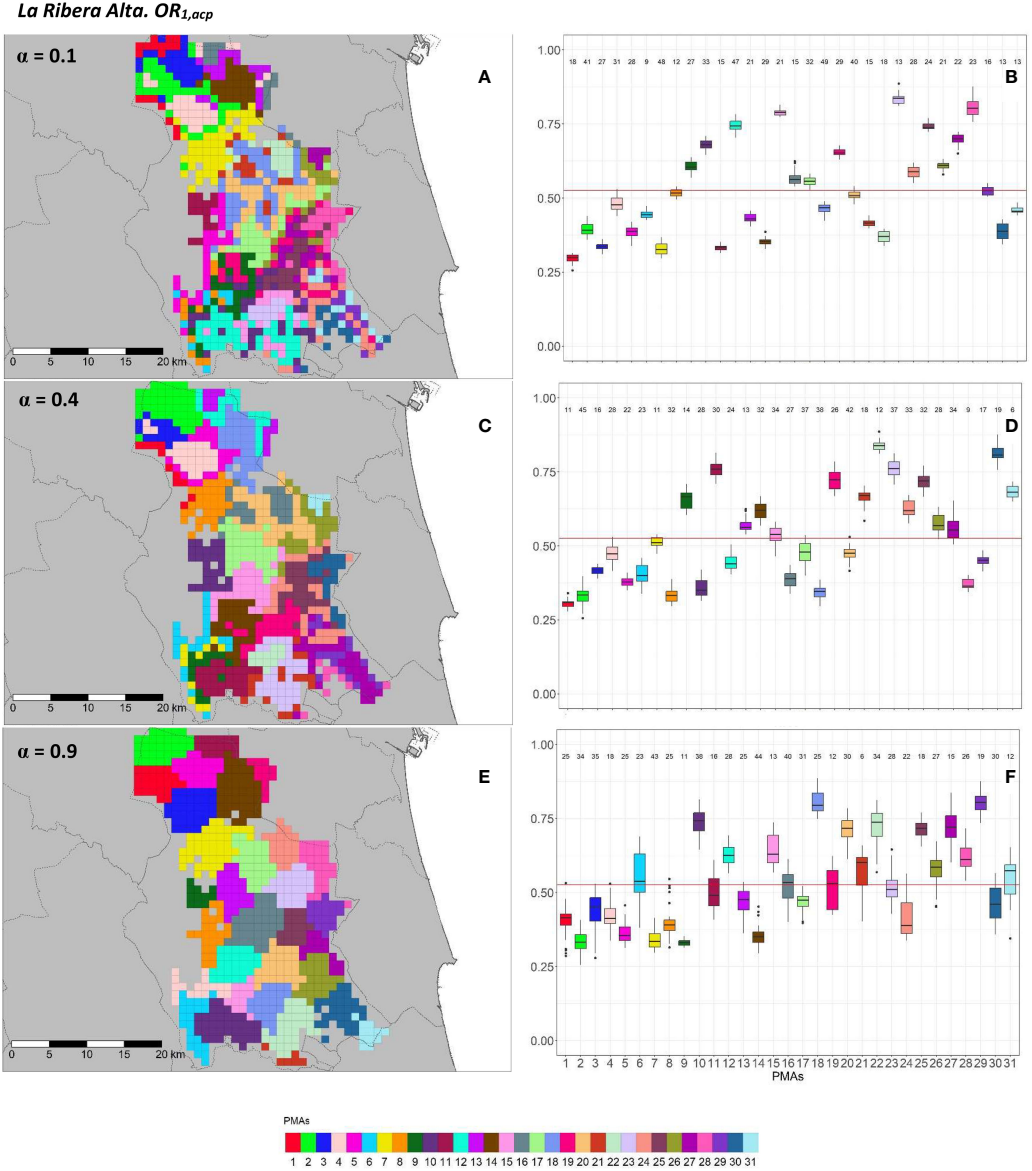
Spatial **(A, C, E)** and overall distribution **(B, D, F)** of the delimited PMAs for *OR*_1_*_,acp_* **(A, C, E)** in “La Ribera Alta” county with *α* of 0.1 **(A, B)**, 0.4 **(C, D)** and 0.9 **(E, F)**. In box-and-whisker plots red line represents the mean value of *OR*_1_*_,acp _* in “El Camp de Túria” county; n quantifies the size of each Pest Management Area in terms of the number of 1 km^2^ cells; central line represents the median value; box limits represent the first (*Q*_1_) and third (*Q*_3_) quantiles; upper whisker represents $min\left(max\left(x\right),Q_{3}+1.5IQR\right)$ ; lower whisker represents $max\left(min\left(x\right),Q_{1}-1.5IQR\right)$ ; $IQR=Q_{3}-Q_{1}$ ; outliers are represented by dots.

**Figure 8:**
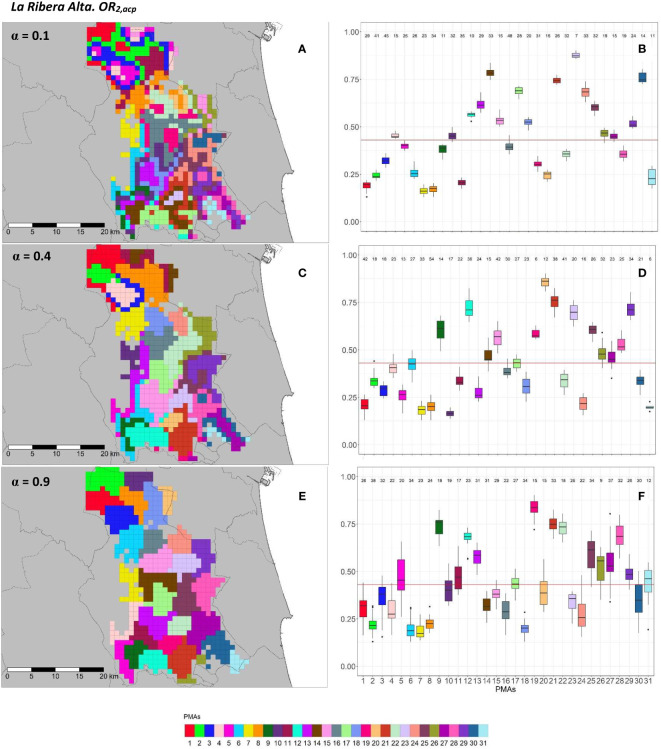
Spatial **(A, C, E)** and overall distribution **(B, D, F)** of the delimited PMAs for *OR*_2_*_,acp_* **(A, C, E)** in “La Ribera Alta” county with *α* of 0.1 **(A, B)**, 0.4 **(C, D)** and 0.9 **(E, F)**. In box-and-whisker plots red line represents the mean value of *OR*_2_*_,acp _* in “El Camp de Túria” county; n quantifies the size of each Pest Management Area in terms of the number of 1 km^2^ cells; central line represents the median value; box limits represent the first (*Q*_1_) and third (*Q*_3_) quantiles; upper whisker represents $min\left(max\left(x\right),Q_{3}+1.5IQR\right)$ ; lower whisker represents $max(min(x),Q_{1}-1.5IQR)$ ; $IQR=Q_{3}-Q_{1}$ ; outliers are represented by dots.

The geographical representation of the sensitivity analysis addressed in the PMA regionalization methodology for ACP on the two overall risks computed (*OR*_1_*_,acp_* and *OR*_2_*_,acp_*) for “El Camp de Túria” and “La Ribera Alta” counties are shown in **Figures 5**, **6** and **Figures 7**, **8**, respectively. Additionally, the internal evaluation measures used to assess the performance of the different values of the *α* parameter are shown in **Table 2**.

**Table 2:** Performance of the regionalization algorithm for the definition of the Pest Management Areas (PMAs) for *Diaphorina citri* (ACP) under the three different specifications of the mixing parameter α for OR_*1,acp*_ and OR_*2,acp*_ in terms of compactness, separation, the Dunn index and the loss of risk homogeneity (1-Q0norm(α)) and increase in spatial aggregation (1-Q1norm(α)) in relation to the reference configurations α = 0 and α = 1, respectively.

County	OR	*α*	Compactness (A)^a^	Separation (B)^b^	Dunn index (D) (B/A)^c^	1-Q_0_norm(*α*)^d^	1-Q_1_ norm(*α*)^e^
El Camp de Túria	$OR_{1,acp}$	0.1	0.157	0.064	0.408	3.6	10.6
		0.2	0.153	0.085	0.556	7.3	3.9
		0.9	0.351	0.126	0.359	25.0	0.2
	$OR_{2,acp}$	0.1	0.127	0.053	0.417	2.4	14.9
		0.3	0.252	0.092	0.365	5.8	6.6
		0.9	0.458	0.150	0.328	24.1	0.4
La Ribera Alta	$OR_{1,acp}$	0.1	0.118	0.046	0.390	1.2	8.0
		0.4	0.148	0.064	0.432	2.6	3.6
		0.9	0.308	0.097	0.315	10.9	0.2
	$OR_{2,acp}$	0.1	0.120	0.058	0.483	1.1	8.2
		0.4	0.196	0.091	0.464	2.4	4.5
		0.9	0.464	0.148	0.319	11.0	0.2

a Estimated by the max(complete.diameter) .

b Estimated by the min(complete.linkage.distance) .

c Estimated as the $D=\frac{min\left(complete.linkage.distance\right)}{max\left(complete.diameter\right)}$ .

d Q_0_ norm(α) denotes the normalized proportion of pseudo-inertias explained by the overall risk.

e Q_1_ norm(α) denotes the normalized proportion of pseudo-inertias explained by the spatial aggregation.

In “El Camp de Túria” county, the number of PMAs was estimated at 25 for both overall risks (*OR*_1_*_,acp_* and *OR*_2_*_,acp_*), according to the restriction on the maximum PMA size (25 cells) imposed in the initial conditions of the algorithm. The value for the *α* parameter that optimized the trade-off between the loss of risk homogeneity and the gain in spatial aggregation was estimated at *α* = 0.2 and *α* = 0.3 under *OR*_1_*_,acp_* and *OR*_2_*_,acp_*. As observed in **Figures 5A, C, E** and **6A, C, E**, higher *α* values are related to a greater increase in the spatial aggregation of PMAs, which is interesting from a practical point of view of the coordinated management of ACP. Nevertheless, they are also the highest *α* values that report the lowest intra-PMA homogeneity as well as the lowest inter-PMAs differences in terms of risk, as shown in **Figures 5B, D, F** and **6B, D, F**. Box-and-whisker plots for each PMA belonging to the *α* = 0.9 present wider distributions and overlap one another (**Figures 5F**, **6F**). The internal validation measures that were estimated supported the graphic results (**Table 2**). Configurations associated with lower *α* values show higher intra-PMAs compactness and PMA separation, and therefore have lower values of the *max*(*complete.diameter*) and *min*(*complete.linkage.distance*), respectively. However, the configurations *α* = 0.2 (for *OR*_1_*_,acp_*) and *α* = 0.1 (for *OR*_2_*_,acp_*) are the ones that maximize the Dunn index, with the best trade-off between compactness and separation. Regarding the loss of risk homogeneity, the *α* = 0.9 configuration resulted in a loss of 25% and 24.1% for *OR*_1_*_,acp _* and *OR*_2_*_,acp_*, respectively, in relation to the *α* = 0 configuration (i.e., 100% risk homogeneity). Thus, they maintain a 75% and 75.9% of risk homogeneity in overall terms. The loss of spatial aggregation for the *α* = 0.9 configuration is 0.2% and 0.6% for *OR*_1_*_,acp_* and *OR*_2_*_,acp_* in relation to the configuration of *α* = 1 (i.e., 100% spatial aggregation).

In “La Ribera Alta” county, the number of PMAs was estimated at 31 for both overall risks (*OR*_1_*_,acp_* and *OR*_2_*_,acp_*), according to the restriction on the maximum PMA size (25 cells) imposed in the initial conditions of the algorithm. The value for the *α* parameter that optimized the trade-off between the loss of risk homogeneity and the gain in spatial aggregation was estimated at *α* = 0.4 for both risks. The influence of the *α* parameter in the performance of the algorithm observed in “El Camp de Túria” was also observed in “La Ribera Alta”. As in “El Camp de Túria”, higher *α* values increased the spatial aggregation of PMAs in “La Ribera Alta” (**Figures 7A, C, E** and **8A, C, E**) but also reduced the intra/inter-PMA risk homogeneity (**Figures 7B, D, F** and **8B, D, F**). Box-and-whisker plots for each PMA with *α* = 0.1 showed the narrowest distributions and lowest overlap (**Figures 7B**, **8B**). The values of the internal validation measures were in line with the graphical results (**Table 2**). Higher intra-PMAs compactness and PMA separation values were obtained with *α* = 0.9, although *α* = 0.4 (for *OR*_1_*_,acp_*) and *α* = 0.1 (for *OR*_2_*_,acp_*) resulted in the best trade-off between compactness and separation with the highest values of the Dunn index (*D* = 0.432 for *OR*_1_*_,acp_* and *D* = 0.483 for *OR*_2_*_,acp_*). In relation to risk homogeneity, *α* = 0.9 resulted in the greatest loss but without exceeding 11% (10.9% for *OR*_1_*_,acp_* and 11% for *OR*_2_*_,acp_*), while resulting in a spatial aggregation of 99.8 (100-0.2)% for both overall risks.

Further description of the individual and overall distribution of the commercial citrus area (km^2^) and number of cells in PMAs in “El Camp de Túria” and “La Ribera Alta” for *OR*_1_*_,acp_* and *OR*_2_*_,acp _* are described in **Supplementary Figures SB1**, **SB2**, respectively.

#### 3.3.2 Trioza erytreae

**Figure 9:**
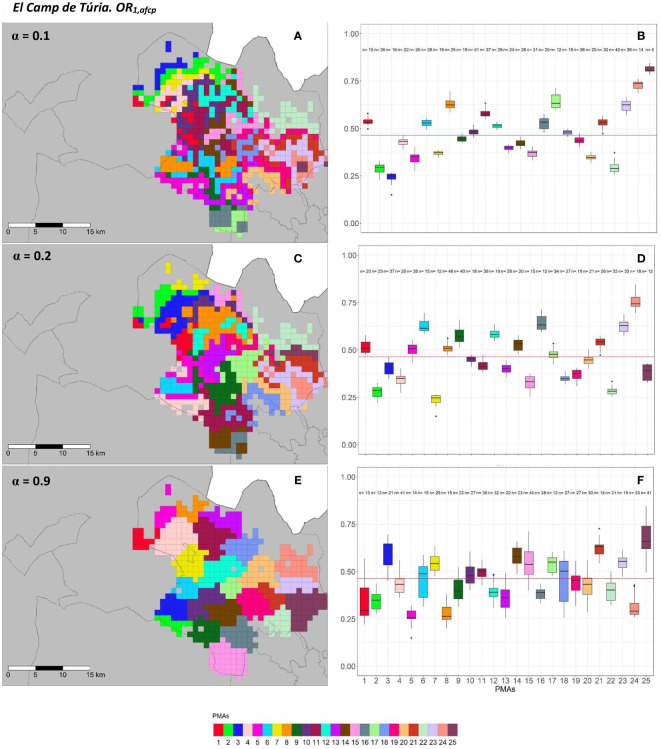
Spatial **(A, C, E)** and overall distribution **(B, D, F)** of the delimited PMAs for *OR*_1_*_,afcp_* **(A, C, E)** in “El Camp de Túria” county with *α* of 0.1 **(A, B)**, 0.3 **(C, D)** and 0.9 **(E, F)**. In box-and-whisker plots red line represents the mean value of *OR*_1_*_,afcp _* in “El Camp de Túria” county; n quantifies the size of each Pest Management Area in terms of the number of 1 km^2^ cells; central line represents the median value; box limits represent the first (*Q*_1_) and third (*Q*_3_) quantiles; upper whisker represents $min\left(max\left(x\right),Q_{3}+1.5IQR\right)$ ; lower whisker represents $max\left(min\left(x\right),Q_{1}-1.5IQR\right)$ ; $IQR=Q_{3}-Q_{1}$ ; outliers are represented by dots.

**Figure 10:**
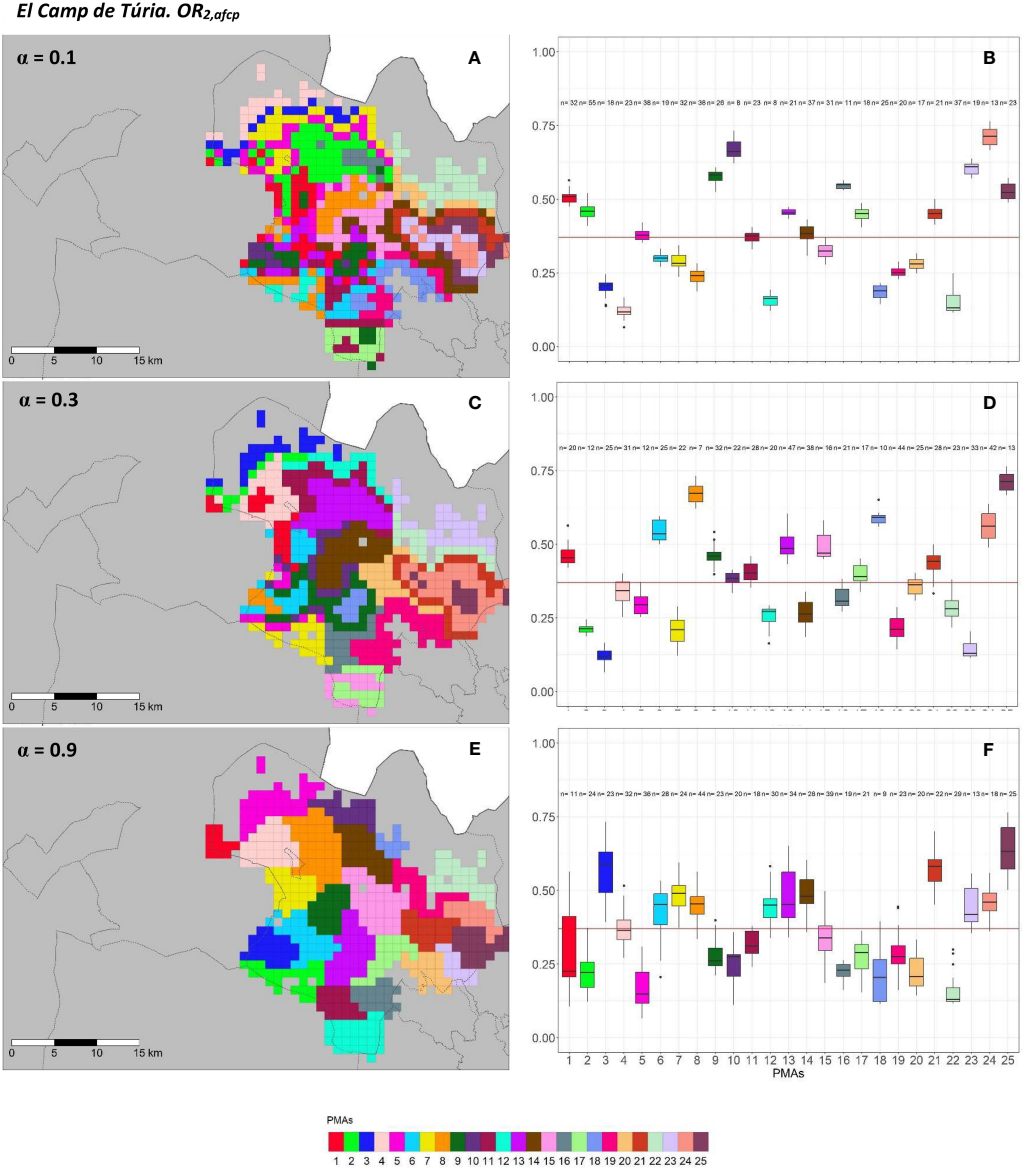
Spatial **(A, C, E)** and overall distribution **(B, D, F)** of the delimited PMAs for *OR*_2_*_,afcp_* **(A, C, E)** in “El Camp de Túria” county with *α* of 0.1 **(A, B)**, 0.3 **(C, D)** and 0.9 **(E, F)**.In box-and-whisker plots red line represents the mean value of *OR*_2_*_,afcp _* in “El Camp de Túria” county; n quantifies the size of each Pest Management Area in terms of the number of 1 km^2^ cells; central line represents the median value; box limits represent the first (*Q*_1_) and third (*Q*_3_) quantiles; upper whisker represents $min\left(max\left(x\right),Q_{3}+1.5IQR\right)$ ; lower whisker represents $max\left(min\left(x\right),Q_{1}-1.5IQR\right)$ ; $IQR=Q_{3}-Q_{1}$ ; outliers are represented by dots.

**Figure 11:**
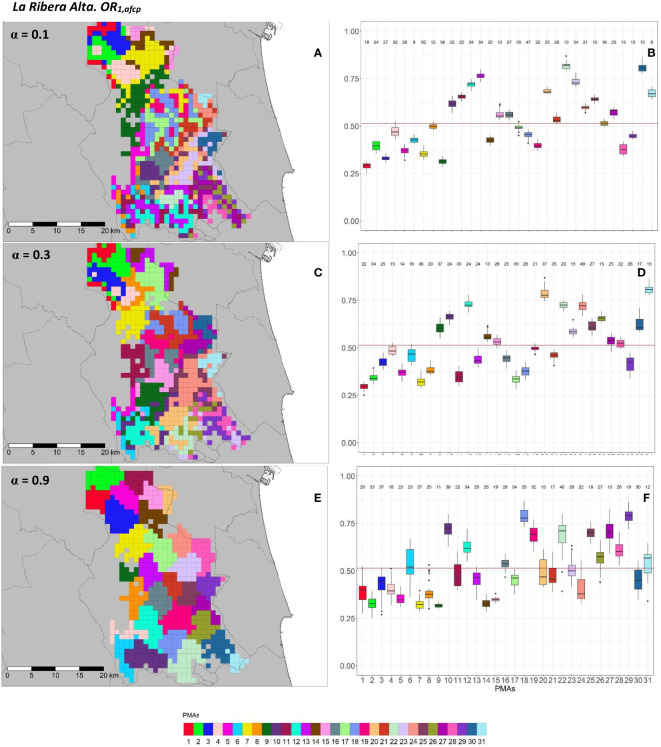
Spatial **(A, C, E)** and overall distribution **(B, D, F)** of the delimited PMAs for *OR*_1_*_,afcp_* **(A, C, E)** in “La Ribera Alta” county with *α* of 0.1 **(A, B)**, 0.4 **(C, D)** and 0.9 **(E, F)**. In box-and-whisker plots red line represents the mean value of *OR*_1_*_,afcp _* in “El Camp de Túria” county; n quantifies the size of each Pest Management Area in terms of the number of 1 km^2^ cells; central line represents the median value; box limits represent the first (*Q*_1_) and third (*Q*_3_) quantiles; quantiles; upper whisker represents $min\left(max\left(x\right),Q_{3}+1.5IQR\right)$ ; lower whisker represents $max\left(min\left(x\right),Q_{1}-1.5IQR\right)$ ; $IQR=Q_{3}-Q_{1}$ ; outliers are represented by dots.

**Figure 12:**
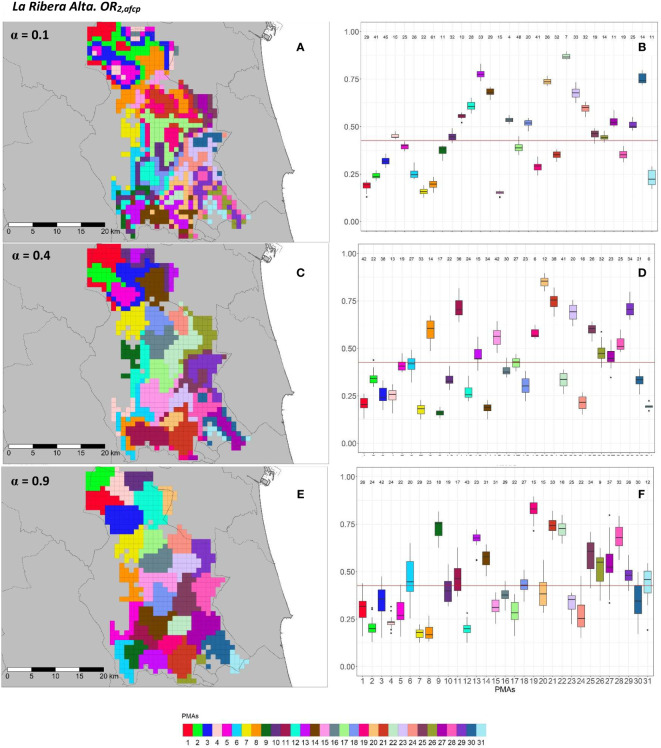
Spatial **(A, C, E)** and overall distribution **(B,D,F)** of the delimited PMAs for *OR*_2_*_,afcp_* **(A, C, E)** in “La Ribera Alta” county with *α* of 0.1 **(A, B)**, 0.4 **(C, D)** and 0.9 **(E, F)**. In box-and-whisker plots red line represents the mean value of *OR*_2_*_,afcp _* in “El Camp de Túria” county; n quantifies the size of each Pest Management Area in terms of the number of 1 km^2^ cells; central line represents the median value; box limits represent the first (*Q*_1_) and third (*Q*_3_) quantiles; upper whisker represents $min\left(max\left(x\right),Q_{3}+1.5IQR\right)$ ; lower whisker represents $max\left(min\left(x\right),Q_{1}-1.5IQR\right)$ ; $IQR=Q_{3}-Q_{1}$ ; outliers are represented by dots.

**Figures 9**, **10** and **Figures 11**, **12** show a graphical overview of the results obtained for AfCP on the two overall risks computed (*OR*_1_*_,afcp_* and *OR*_2_*_,afcp_*) for “El Camp de Túria” and “La Ribera Alta” counties. The internal evaluation measures used to assess the performance of the different choices of the *α* parameter are displayed in **Table 3**.

**Table 3:** Performance of the regionalization algorithm for the definition of the Pest Management Areas (PMAs) for *Trioza erytreae* (AfCP) under the three different specifications of the mixing parameter *α* for OR_*1,afcp*_ and OR_*2,afcp*_ in terms of compactness, separation, the Dunn index and the loss of risk homogeneity (1-Q_0_ norm(*α*)) and increase in spatial aggregation (1-Q_1_ norm(*α*)) in relation to the reference configurations *α* = 0 and *α* = 1, respectively.

County	OR	*α*	Compactness (A)^a^	Separation (B)^b^	Dunn index (D) (B/A)^c^	1-Q_0_ norm(*α*)^d^	1-Q_1_ norm(*α*)^e^
El Camp de Túria	$OR_{1,afcp}$	0.1	0.128	0.060	0.469	3.1	11.8
		0.2	0.151	0.087	0.576	7.2	4.2
		0.9	0.351	0.140	0.399	27.6	0.1
	$OR_{2,afcp}$	0.1	0.134	0.067	0.500	2.4	15.6
		0.3	0.172	0.102	0.593	5.6	6.8
		0.9	0.457	0.147	0.322	20.4	0.7
La Ribera Alta	$OR_{1,afcp}$	0.1	0.094	0.058	0.617	1.2	8.2
		0.3	0.134	0.067	0.500	2.6	3.5
		0.9	0.407	0.078	0.192	11.7	0.2
	$OR_{2,afcp}$	0.1	0.119	0.060	0.504	1.1	8.3
		0.4	0.198	0.092	0.465	2.4	4.4
		0.9	0.463	0.142	0.307	11.1	0.2

a Estimated by the max(complete.diameter) .

b Estimated by the min(complete.linkage.distance) .

c Estimated as the $D=\frac{min\left(complete.linkage.distance\right)}{max\left(complete.diameter\right)}$

d Q_0_ norm(α) denotes the normalized proportion of pseudo-inertias explained by the overall risk.

e Q_1_ norm(α) denotes the normalized proportion of pseudo-inertias explained by the spatial aggregation.

In “El Camp de Túria” county the number of PMAs was estimated at 25 for both overall risks (*OR*_1_*_,afcp_* and *OR*_2_*_,afcp_*), according to the restriction on the maximum PMA size (25 cells) imposed in the initial conditions of the algorithm. The value that optimized the trade-off between the loss of risk homogeneity and the gain in spatial aggregation was *α* = 0.2 for *OR*_1_*_,afcp_* and *α* = 0.3 for *OR*_2_*_,afcp._* **Figures 9**, **10** show that higher *α* values increased the spatial aggregation of PMAs (**Figures 9E**, **10E**) but resulted in less compact and more separated PMAs in terms of risk homogeneity (**Figures 9F**, **10F**). Box-and-whisker plots for each PMA with *α* = 0.9 resulted in wider distributions and a higher overlap (**Figures 9F**, **10F**). Lower *α* values resulted in higher intra-PMAs compactness and PMA separation, with lower values of *max*(*complete.diameter*) and *min*(*complete.linkage.distance*) (**Table 3**). Nevertheless, the highest values of the Dunn index were obtained with *α* = 0.2 for *OR*_1_*_,afcp_* and *α* = 0.3 for *OR*_2_*_,afcp._* In relation to risk homogeneity, *α* = 0.9 resulted in the greatest loss but not exceeding 27.6% (27.6% for *OR*_1_*_,afcp_* and 20.4% for *OR*_2_*_,afcp_*) in comparison to *α* = 0, while it had a spatial aggregation of 99.9 (100-0.1)% and 99.3 (100-0.7)%, in comparison to the configuration of *α* = 1, for *OR*_1_*_,afcp_* and *OR*_2_*_,afcp,_* respectively.

In “La Ribera Alta” county, the number of PMAs was estimated at 31 for both overall risks (*OR*_1_*_,afcp _* and *OR*_2_*_,afcp_*), according to the restriction on the maximum PMA size (25 cells) imposed in the initial conditions of the algorithm. Values of *α* = 0.3 for *OR*_1_*_,afcp_* and *α* = 0.4 for *OR*_2_*_,afcp_* resulted in the best trade-off between the loss of risk homogeneity and the gain in spatial aggregation. **Figures 11**, **12** show that higher *α* values increased spatial aggregation but reduced the intra/inter-PMAs compactness and separation in terms of risk homogeneity. Box-and-whisker plots for each PMA with *α* = 0.1 showed the narrowest distributions with the lowest overlap (**Figures 11B**, **12B**). Higher intra-PMAs compactness and PMA separation values were obtained with *α* = 0.9, although *α* = 0.1 showed the best trade-off between compactness and separation with the highest value of the Dunn index (*D* = 0.617 for *OR*_1_*_,afcp _* and *D* = 0.504 for *OR*_2_*_,afcp_*) (**Table 3**). In relation to risk homogeneity, *α* = 0.9 resulted in the highest reduction, 11.7% for *OR*_1_*_,afcp_* and 11.1% for *OR*_2_*_,afcp _* but resulted in a spatial aggregation of 99.8 (100-0.2)% for both risks.

Further description of the individual and overall distribution of the commercial citrus area (km^2^) and number of cells in PMAs in “El Camp de Túria” and “La Ribera Alta” for *OR*_1_*_,afcp_* and *OR*_2_*_,afcp_* are described in **Supplementary Figures SB3** and **SB4**, respectively.

## 4 Discussion

This study presents a risk-based approach to defining management areas for HLB vectors. The rationale of this approach is based on the regionalization of citrus-growing areas into Pest Management Areas (PMAs) that share a similar overall risk to the introduction and spread of insect vectors but considering spatial constraints to make it feasible from the viewpoint of implementation. The size and location of PMAs were estimated using the Ward-like hierarchical algorithm developed by Chavent et al. (2018). This spatial aggregation algorithm provides relevant elements for the grouping of areas under similar risk and allows the incorporation of geographical information to address the clustering process, which in the context of vector-borne plant diseases is a fundamental point to support the operational implementation (Bassanezi et al., 2013; Yuan et al., 2021). The Valencian Autonomous Community (ES52 NUTS 2), Spain, was selected as the study area because it is the largest citrus-growing region in Europe and is representative of others in the Mediterranean Basin. Specifically, PMAs were designed and optimized for “La Ribera Alta” and “El Camp de Túria” counties.

To date, several proposals for area-wide management of HLB have been developed, framed in different epidemiological settings  (Bové, 2006; Manjunath et al., 2010; Kumagai et al., 2013; Flores-Sánchez et al., 2017; Badaracco et al., 2021; García-Figuera et al., 2021a). In general, the rationale behind these approaches considers regional risk factors, estimated based on epidemiological variables such as cultivar susceptibility, host density, and vector presence and abundance (Gottwald et al., 2014a; Flores-Sánchez et al., 2017). Some of them also incorporate other factors related to human factors, such as the availability of infrastructure, human and economic resources in the citrus-producing regions, as in Mexico (Flores-Sánchez et al., 2017), or the social willingness to participate, as in California (McRoberts et al., 2019; García-Figuera et al., 2022). Nevertheless, although previous research has shown improved HLB control with the area-wide strategy (Bassanezi et al., 2013; García-Figuera et al., 2021a), the standard settings for determining the size and location of area-wide management have not been established (Flores-Sánchez et al., 2017).

Also, different recommendations have been suggested concerning the area-wide size to manage HLB. Using empirical data, Bové (2012) proposed an area size of 500 ha in Brazil, whereas sizes in California range from 4,000 to 20,000 ha (Flores-Sánchez et al., 2017). Nonetheless, the farming systems in Brazil and California are usually composed of large orchards. This contrasts with the citrus-growing areas in the Mediterranean Basin like Spain, where farms are smaller and divided up into orchards of <1 ha often managed independently by individual growers (Fernández-Zamudio et al., 2006). Thus, PMAs aggregation will probably be more challenging from an extension point of view.

To implement our proposal, the study area was characterized by several risk factors at a grid resolution of 1 km^2^ using data on climatic suitability, conventional, organic, abandoned and residential citrus areas, and transportation corridors. These risk factors have been associated with the introduction and spread of ACP, and in consequence with HLB epidemiology (Halbert et al., 2010; Richards et al., 2013; Gottwald et al., 2014a; Martini et al., 2015; Bayles et al., 2017; Flores-Sánchez et al., 2017). Our proposal considers the same approach for AfCP. While there have been extensive studies of ACP and AfCP in other regions, there is limited knowledge about both vectors’ biology, spread capacity and host range in the Mediterranean Basin. Likewise, the Valencian Autonomous Community remains free from HLB and from both ACP and AfCP and so it is uncertain how the vectors and the disease will behave.

The risk factors included in our study do not consider the wind effects (i.e., direction and speed) although previous studies on ACP spread dynamics and area-wide management often consider this factor to predict vector distribution in an invading scenario from an entry point. Antolínez et al. (2022) indicated that ACP adults are likely to spread across the prevailing wind direction and that wind speeds higher than 48 km/h are able to dislodge adults from citrus leaves and trigger passive long-distance spread events. Moreover, some studies have hypothesized that severe meteorological wind-related episodes, such as hurricanes in Florida, greatly influenced the major long-distance spread of ACP in Florida (Johnston et al., 2019; Monzó and Vanaclocha, 2023). Nevertheless, the availability of accurate wind data at large scales on a regional resolution is scarce given that wind observations vary on small space and time scales due to the fact that wind is affected by the local terrain, vegetation and buildings. Furthermore, recent studies have shown that other factors, such as orchard layout, have a stronger influence on HLB incidence and spread than wind (Benhadi-Marín et al., 2021; Primiano et al., 2023). Other risk factors integrated in previous studies, such as packing houses and nurseries (Gottwald et al., 2014a; Flores-Sánchez et al., 2017), were not included either. The relevance of these factors in the vector distribution also remains unclear. Similarly, host data (variable and cultivar) was not included because of the lack of available data on cultivar preference and susceptibility.

Nevertheless, the proposed regionalization methodology for estimating the size and location of PMAs ensures sufficient flexibility to update the default risk factors considered or to add new ones as more information about the insect vectors/disease become available or as the epidemiological setting evolves. Similarly, although in our study two options have been proposed for estimating the overall risk, i) averaging its influence, and 2) giving greater importance to the risk posed by the presence of abandoned and organic orchards than to the rest of the risks, other criteria can be accommodated. In the same way, further refinements may be implemented for the risk factor normalization in the presence of extreme values.

The approach proposed in our study for defining the size and location of PMAs makes use of a hierarchical clustering algorithm that allows the introduction of spatial constraints (Chavent et al., 2018). This kind of clustering methodology is usually called regionalization. The term regionalization was defined by Guo (2008) as the process of aggregating a set of spatial entities into a reduced number of regions in a way that a predefined objective function is optimized. Spatial contiguity and homogeneity are the basic criteria underpinning regionalization. Spatial contiguity requires that spatial connectivity or spatial tightness should be met. Beyond the ClustGeo (Chavent et al., 2018), there are several important regionalization algorithms such as SKATER (Assunção et al., 2006) or REDCAP (Guo, 2008). However, ClustGeo was chosen because it enables the definition of the spatial relationship between the units intended for grouping through the assessment of similarity between geographical coordinates, without the necessity for these units to satisfy explicit spatial connectivity requirements, as, for instance, demanded by SKATER (Assunção et al., 2006). This functionality offered by ClustGeo was the decisive factor that led us to opt for this algorithm, given the specific spatial arrangement characteristics of our study area concerning the units to be grouped.

These regionalization techniques are used in several disciplines, including sociology, economics, urban planning, politics and health to identify areas with similar characteristics and to get useful information to support policy-making (Camêlo Aguiar et al., 2020; Guo et al., 2022). However, to our knowledge, this is the first time that this methodology has been used in plant health to group areas with a similar level of risk of vector introduction and spread in order to address a coordinated management and, additionally, on an open-source implementation. The greatest potential of this algorithm in the frame of area-wide management is that it makes it possible to consider the spatial attributes of the units to be clustered and to modulate the importance of spatial aggregation versus the “risk” in the design of the solutions. This importance of spatial aggregation is defined in the implementation offered (i.e., the R package ClustGeo) through the setting of the *α* parameter. Specifically, when *α* = 0 the spatial dissimilarities are not taken into account and when *α* = 1 the “non-spatial attributes” distances are not considered and only the spatial distances are taken into account (Chavent et al., 2018; Camêlo Aguiar et al., 2020).

The Clustgeo algorithm presents a marked variability in the design of the results depending on the setting of parameter *k* (i.e., maximum number of clusters or PMAs allowed) and depending on the value of parameter *α*. Specifically, the choice of the maximum number of PMAs (i.e., clusters) was defined assuming a maximum PMA size of 25 cells based on expert criteria, but this parameter can be adjusted by the end user or optimized based on different criteria. Furthermore, a sensitivity analysis was performed to evaluate the effect of three different values of *α*. For this purpose, in addition to the graphical results, several internal validation measures were computed to quantify compactness and/or separation as well as the trade-off between the loss of risk homogeneity and the loss of spatial aggregation (Leskovec et al., 2014; Kassambara, 2017). For *α* = 0.9 under the four configurations evaluated (O*R*_1_*_,acp_*, O*R*_2_*_,acp_*, O*R*_1_*_,afcp_*, O*R*_2_*_,afcp_*), the results obtained in both counties, “La Ribera Alta” and “El Camp de Túria”, showed the greatest spatial aggregation in the design of the location of PMAs, which is fundamental for their implementation from a practical point of view. Furthermore, with *α* = 0.9 the solutions also supported at least 72.4% homogeneity in terms of overall risk.

In sum, this PMAs design proposal, addressing two overall risk estimates for both ACP and AfCP, offers a comprehensible, accessible and adaptable approach both to estimating the risk factors associated with the introduction and spread of the two HLB vectors and to establishing their corresponding management areas (PMAs). Although it was implemented at NUTS2 level due to administrative reasons, this framework can be upscaled at country and even at Mediterranean Basin levels to implement a standard management and minimize the possible migration of vectors among areas. Thus, the development of this PMAs design proposal means having a preventive tool at the service of the citrus industry. Its use can be extended beyond the coordination of vector/disease management, for instance, to the field of epidemiological surveillance as well as to the context of education and awareness-raising.

Nevertheless, future work would be needed to verify the citrus data at orchard level and its classification as conventional, organic, abandoned, residential, etc. Indeed, the locations of organic and abandoned citrus orchards were randomly allocated to reach the total area reported by Ministerio de Agricultura, Pesca y Alimentación (MAPA) (2019; 2020). The isotherm for citrus growth and population census data were used to estimate the residential citrus areas. Thus, it would also be necessary to update the default risk factors considered, and to add new ones when more information about the insect vectors/disease become available after an eventual introduction in the study area. Additionally, social willingness toward the adoption of PMAs should be properly addressed. Previous experiences from regions with the presence of disease and vector have demonstrated that the only successful action to control the spread of HLB is to control the vector on a large spatial scale (Singerman et al., 2017; Milne et al., 2018; Alquézar et al., 2022). Area-wide programs in countries with the presence of HLB typically rely on voluntary adoption (García-Figuera et al., 2021a; García-Figuera et al., 2022). To achieve voluntary adoption, in our case, the advantages of the coordinated treatments should be effectively communicated and make stakeholders trust the risk manager to keep them motivated and involved in the program (Klassen, 2008; Singerman et al., 2017; McRoberts et al., 2019; Monzó and Stansly, 2020; García-Figuera et al., 2021b; Pavone, 2022).

## Data availability statement

The original contributions presented in the study are included in the article/**Supplementary Material**. Further inquiries can be directed to the corresponding author. Data curation and formal analysis have been implemented in the R statistical programming. All the generic code used in the research is open-source and available at https://zenodo.org/records/10079629.

## Author contributions

AG: Data curation, Writing – original draft, Writing – review & editing. RB: Conceptualization, Writing – review & editing. WL: Conceptualization, Writing – review & editing. PV: Methodology, Writing – original draft, Writing – review & editing. AV: Conceptualization, Data curation, Funding acquisition, Methodology, Project administration, Supervision, Writing – original draft, Writing – review & editing. EL: Data curation, Writing – original draft, Conceptualization, Formal analysis, Investigation, Methodology, Software, Writing – review & editing.

## Supplementary material

Supplementary Material A Climatic conditions, landscape architecture and citrus industry practices affecting introduction and spread of citrus psyllids.

Supplementary Material B Pest Management Areas (PMAs) descriptive.
