# Supplementary material for: Risk-based regionalization approach for area-wide management of HLB vectors in the Mediterranean Basin
Source: Front Plant Sci. 2023 Dec 4;14:1256935. doi: 10.3389/fpls.2023.1256935 (PMC10725980; doi:10.3389/fpls.2023.1256935)
Supplement: Supplementary Material A — Climatic conditions, landscape architecture and citrus industry practices affecting introduction and spread of citrus psyllids. [file DataSheet_1.pdf]

Supplementary Material A. Climatic conditions, landscape architecture and citrus industry practices affecting the introduction and spread of citrus psyllids.

The populations, distribution, abundance, establishment and spread of the Asian citrus psyllid, *Diaphorina citri*, and the African citrus psyllid, *Trioza erytreae* are influenced by biotic, abiotic and anthropogenic factors. Among them, natural enemy regulation, climatic conditions, landscape architecture (host plant availability, phenological stage of host plants, and crop management strategies) and citrus industry practices are the ones that most affect the biology of these citrus psyllids. The effects of these factors nevertheless vary depending on the region, local conditions and the species of psyllid involved. Understanding these effects is essential to predict vector dispersal and to implement and develop effective management strategies that minimize the impact of huanglongbing (HLB).

1 CLIMATIC CONDITIONS

As with most insects, citrus psyllids are strongly influenced by climatic conditions. Climatic variables limit the presence and abundance of these species. **Temperature (T)** and **relative humidity (RH)** are probably the most relevant variables in insect biology, so understanding their effects is crucial to interpret how climate affects the risk of establishment and spread capability.

The temperature helps to predict life cycle duration and development potential. Unfavorable temperatures can limit establishment and abundance (Catling, 1969b; Catling and Green, 1972; Cocuzza et al., 2017; Monzó and Vanaclocha, 2023). There are two important temperature thresholds that are commonly used to understand the thermal biology of insects and to model and predict their population dynamics:

(i) Lower threshold temperature (T_l): this is the minimum temperature at which an insect can complete its development. Below this temperature, the insect has very limited activity and can find it difficult to develop.

(ii) Upper threshold temperature (T_u): this is the highest temperature at which the insect can develop optimally. At temperatures above this threshold, development is negatively affected and may experience a significant reduction in survival rate.

The relative humidity (RH) can also influence the development of citrus psyllids. It has been observed that high relative humidity conditions favor the development and survival of psyllids. On the other hand, low humidity conditions are less favorable and negatively impact their development. The effects of RH on insect biology are usually mediated by temperatures. In the case of citrus psyllids, negative impacts of low RH environments are magnified when combined with high temperatures. The saturation deficit (SD) is a variable that includes both temperature and relative humidity and has been used in the past to understand the biological requirements of *T. erytreae* (Catling, 1969b). The SD is a measure of the drying potential of the atmosphere and it is defined as the difference between the actual water vapor pressure and the saturated vapor pressure at a given temperature. High SD values reduce the survival rates of this psyllid significantly. Additionally, there are other climatic variables that interact and affect the survival,

distribution, movement and adaptation of citrus psyllids, such as rain, light and wind, and can determine their activity and preference for certain habitats.

Another important climatic parameter to consider in insect thermal biology is the degree days (DD). This is defined as the accumulated amount of heat required for an egg to become an adult and therefore complete their development. The lower threshold temperature (T_l; also named as base temperature) and the degree days (DD) are used to explain how insect establishment, development and reproduction depend on temperature (Honěk et al., 1990) and to estimate the potential number of generations (PNG).

The thresholds of these climatic variables above or below which species cannot develop can be used to define a favorable day for development (FD). T_l and T_u can be used for a definition of FD and sometimes RH or SD thresholds can also be considered.

Specific climatic requirements of *Diaphorina citri* and *Trioza erytreae*

Although many species of Psyllidae are known to be intolerant to relatively high temperatures, *D. citri* is well adapted to high thermal conditions (Catling, 1969b; Catling and Green, 1972; Cocuzza et al., 2017; Monzó and Vanaclocha, 2023). It is able to develop at mean temperatures between 10°C, as a T_l, and 33°C, as a T_u(Liu and Tsai, 2000). For a *D. citri* egg to become an adult, Liu and Tsai (2000) estimated a thermal requirement of 249.88 DD and a specific T_l of 10.45°C. *D. citri* thermal requirements make this species better adapted to tropical and subtropical climates and hot, coastal zones (Catling et al., 1970; Hodkinson, 2009; Jenkins et al., 2015). *D. citri* is also well adapted to dry conditions. Consequently, this insect species is able grow even in citrus cultivated in tropical arid climates such as that of the Arabian Peninsula (Bové, 2006).

In contrast, *T. erytreae* prefers cool moist conditions and it hardly develops under hot dry climates (Catling, 1969b) or even in climates characterized by high temperatures and frequent rain (Catling, 1969a; Green and Catling, 1971; Tamesse and Messi, 2004). *T. erytreae* is able to develop at mean temperatures between 10°C, as T_l, and 27°C, as T_u (Aidoo et al., 2022). Within this temperature range, the insect's life cycle progresses efficiently, facilitating population growth and establishment in suitable environments. While *T. erytreae* thrives within the aforementioned temperature range, it is especially sensitive to the combination of high temperatures and low RH. Studies conducted by Catling (1969b, 1972) on the biology of this insect revealed the significance of SD values in determining its survival thresholds. When SD values reach more than 32.1 mm Hg, *T. erytreae* experiences considerable mortality rates (Catling, 1969a). The estimated thermal requirements for an egg to become an adult are 270 DD with a T_l that lies between 10 and 12°C (Catling, 1973; Aidoo et al., 2022)

2 LANDSCAPE ARCHITECTURE

Host plant availability and phenological stage

Diaphorina citri and *T. erytreae* primarily infest plants in the Rutaceae family, preferably *Citrus* spp. However, they can also feed on other Rutaceae family members, including some ornamental plant species (Grafton-Cardwell et al., 2013; Cocuzza et al., 2017; Hall, 2020; Monzó and Vanaclocha, 2023). The availability and distribution of citrus plants play a significant role in determining the populations and establishment of both species. The presence of suitable host plants contributes to their abundance and spread. In addition to the agricultural citrus landscapes, citrus psyllids can also be found in residential areas, which must therefore also be considered as risk areas for unchecked psyllid populations proliferation.

Psyllids present in these areas can move between residential landscapes and nearby commercial citrus orchards, increasing the risk of infestation and establishment.

Citrus psyllid generations are conditioned by the phenological stages of citrus trees. The availability of emergent leaves (leaf flushes) and tender young leaves provides a suitable environment for the psyllids to feed, reproduce and develop, and determines the psyllid population (Catling, 1969a, 1972; Catling and Green, 1972; Cocuzza et al., 2017; Cifuentes-Arenas et al., 2018; Hall, 2020; Monzó and Vanaclocha, 2023). Changes in host phenology can result in fluctuations in the population size and spatial distribution of the psyllids. Understanding the relationship between citrus host phenology and the populations of the citrus psyllids is essential to implement effective management strategies.

Diaphorina citri and *T. erytreae* gravid females lay eggs on leaf flushes, sometimes including young leaves associated with emergent floral shoots (Cifuentes-Arenas et al., 2018). Immature stages only feed on tender tissue (unhardened leaves) of the growing shoots and they cannot develop on mature leaves. Survival of the psyllids consequently depends on the availability of soft plant tissues (Annecke and Cilliers, 1963; Moran and Buchan, 1975; van den Berg and Deacon, 1988; Hall, 2020; Monzó and Vanaclocha, 2023). Young citrus trees present more frequent and prolonged growing periods, which makes them a preferred host for gravid females (Croxton and Stansly, 2014).

Citrus trees grown in humid tropical or subtropical regions typically have overlapping phenological stages throughout the year with multiple flushing periods in which psyllids can develop (Udell et al., 2017). Under Mediterranean or semi-arid conditions, the number and intensity of flushing periods is limited and especially influenced by factors such as temperature, rainfall, and tree irrigation, pruning, and nutrition. In the Mediterranean Basin, citrus-growing areas commonly experience three flushing periods that typically coincide with favorable weather conditions and the natural growth cycles of citrus trees: i) spring flush, which is usually the most significant and occurs typically from late February to May. It follows the winter dormancy period and, as temperatures rise, new growth emerges, characterized by the development of new leaves, shoots, and flowers; ii) summer flush, when suitable weather conditions are given at the end of spring, a second flush period may occur typically between June and August. It is usually less vigorous than the spring flush but can contribute to new growth and fruit development; and iii) fall flush, which usually occurs from September to November following humid weather at the end of the summer (Hermoso De Mendoza et al., 2012; Lebbal and Laamari, 2016; Bouvet et al., 2019; Garcia-Marí et al., 2002).

The development stages of citrus psyllids exhibit an aggregated behavior on young shoots, where egg laying and immature development occur while adult psyllids are known to move frequently between orchards (Samways and Manicom, 1983; Sétamou et al., 2008). For *D. citri*, it has been observed that citrus flushes overcrowded with nymphs competing for the same food resource trend to trigger spread behaviors within and among citrus orchards (Chiyaka et al., 2012; Udell et al., 2017).

Crop management practices

Development of effective management strategies for insect pests requires an understanding of their spatial distribution patterns within the agricultural landscape (Hall and Gottwald, 2011). Mediterranean citrus landscapes encompass conventional, organically managed, and abandoned orchards. Each type of orchard has its particularities and consequently presents unique challenges and opportunities for controlling and preventing the spread of *D. citri*. While conventional orchards may rely on chemical pesticides for effective citrus psyllids management, organic management emphasizes natural and biological control methods and, consequently, controlling the populations of citrus psyllids can be more challenging. Abandoned fields may act as reservoirs of citrus psyllids where their population can proliferate unchecked (Tiwari et al., 2010).

In humid subtropical regions, it has been demonstrated that the abundance of citrus psyllids in abandoned orchards is higher than in orchards with conventional citrus management and this may contribute to the spread of these vectors (Sétamou and Bartels, 2015; Martini et al., 2016). However, their role in the spread of vectors may be somehow limited by the lower growth activity of citrus trees in those fields, especially in semi-arid citrus-growing regions such as those with Mediterranean climates.

The activity by which *D. citri* spread has been associated with the availability and quality of food resources (Sétamou et al., 2008; Udell et al., 2017) and with environmental factors (Martini and Stelinski, 2017; Tomaseto et al., 2018). *Diaphorina citri* can disperse over distances of at least 2 km (Lewis-Rosenblum et al., 2015). There is evidence of rapid short-distance movement of *D. citri* between unmanaged (abandoned) and managed orchards (Boina et al., 2009). It has been reported that *T. erytreae* is able to disperse up to 1.5 km with females moving longer distances than males (van den Berg and Deacon, 1988).

3 CITRUS INDUSTRY PRACTICES

The spread distance of these psyllid species can also be influenced by human activities, such as the transportation of infested plant material, the movement of citrus trees or even harvested fruits (Halbert et al., 2010). Infested young citrus material is known to be one of the potential sources of spread for *D. citri* (Halbert and Manjunath, 2004). Trailers transporting citrus fruit from orchards to packing houses were recognized as a source of *D. citri* spread (Halbert et al., 2010). The rutaceous plant, *Murraya paniculata*, used as an ornamental bush or hedge, is one of the preferred hosts of *D. citri* and can carry *D. citri* eggs or nymphs. The entry of this or other host plants into a ‘clean’ region is therefore associated with a risk of introducing *D. citri* (Halbert and Manjunath, 2004). Additionally, the availability of suitable host plants in the surrounding landscape can affect their spread patterns. Transport corridors provide a linear pathway for citrus psyllids to disperse, allowing them to move efficiently from one location to another. This can contribute to the rapid spread of these pests, especially if suitable host plants are present along the transport routes. Agricultural practices within the citrus industry, including machinery and equipment, can facilitate the spread of these psyllids.

4 ESTIMATION OF THE POTENTIAL NUMBER OF GENERATIONS AND FAVORABLE DAYS WITHIN THE STUDY AREA

The potential number of generations per year (PNG) for each cell i was calculated based on the average cumulative degree-days for the 10-year period (2009-2018) and dividing by the degree-days (DD) required for an egg of *D. citri* / *T. erytreae* to become an adult above a developmental threshold temperature (T_l). For *D. citri*, $T_l = 10.45$°C and $DD = 249.88$°C (Liu and Tsai, 2000). For *T. erytreae*, $T_l = 10$°C and $DD = 270.00$°C (Catling, 1973; Aidoo et al., 2022).

The number of favorable days (FD) for each cell i was calculated considering only those days allowing the development of all the stages of the insect. For *D. citri*, “a favorable day” was defined considering that this insect species is able to develop with mean temperatures (T_{mean}) between 10°C, as a lower threshold (T_l), and 33°C, as an upper threshold (T_u) (Liu and Tsai, 2000). For *T. erytreae* a “favorable day” was defined considering that it is able to develop at mean temperatures (T_{mean}) between 10°C, as T_l, and 27°C, as T_u (Aidoo et al., 2022) and also including a saturation deficit (SD) $\leq$ 32.1 mmHg (Catling, 1969b, 1972).

For PNG and FD estimation daily climate variables were filtered considering only the days from 15 February to 30 April, June and October, assuming that under Mediterranean conditions these are the three major leaf flushing periods (Garcia-Marí et al., 2002).

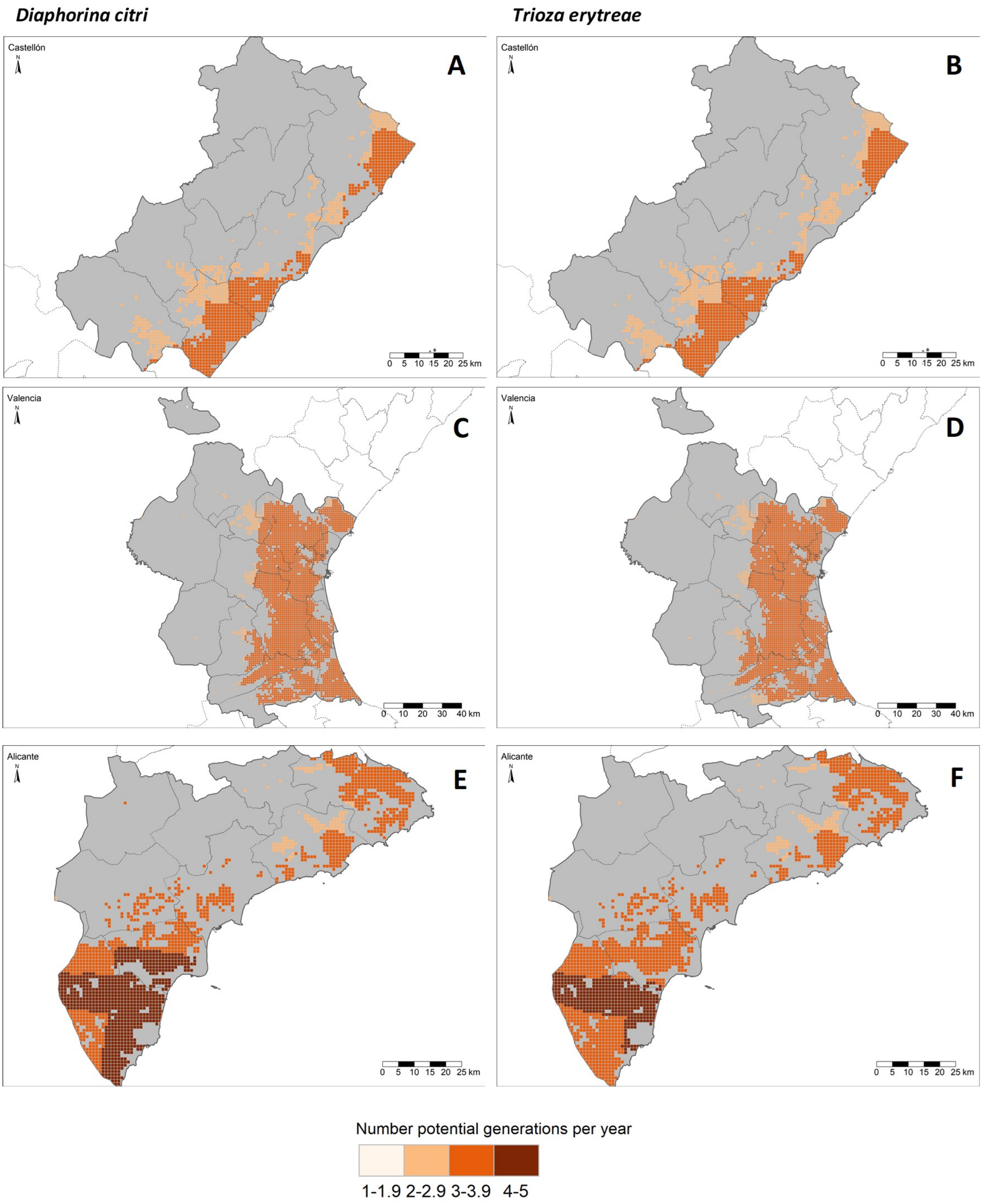

Figure SA1. Potential number of generations per year estimated for *Diaphorina citri* (A,C,E) and *Trioza erytreae* (B,D,F) disaggregated at province level (NUTS 3): Castellón (A,B), Valencia (C,D) and Alicante (E,F).

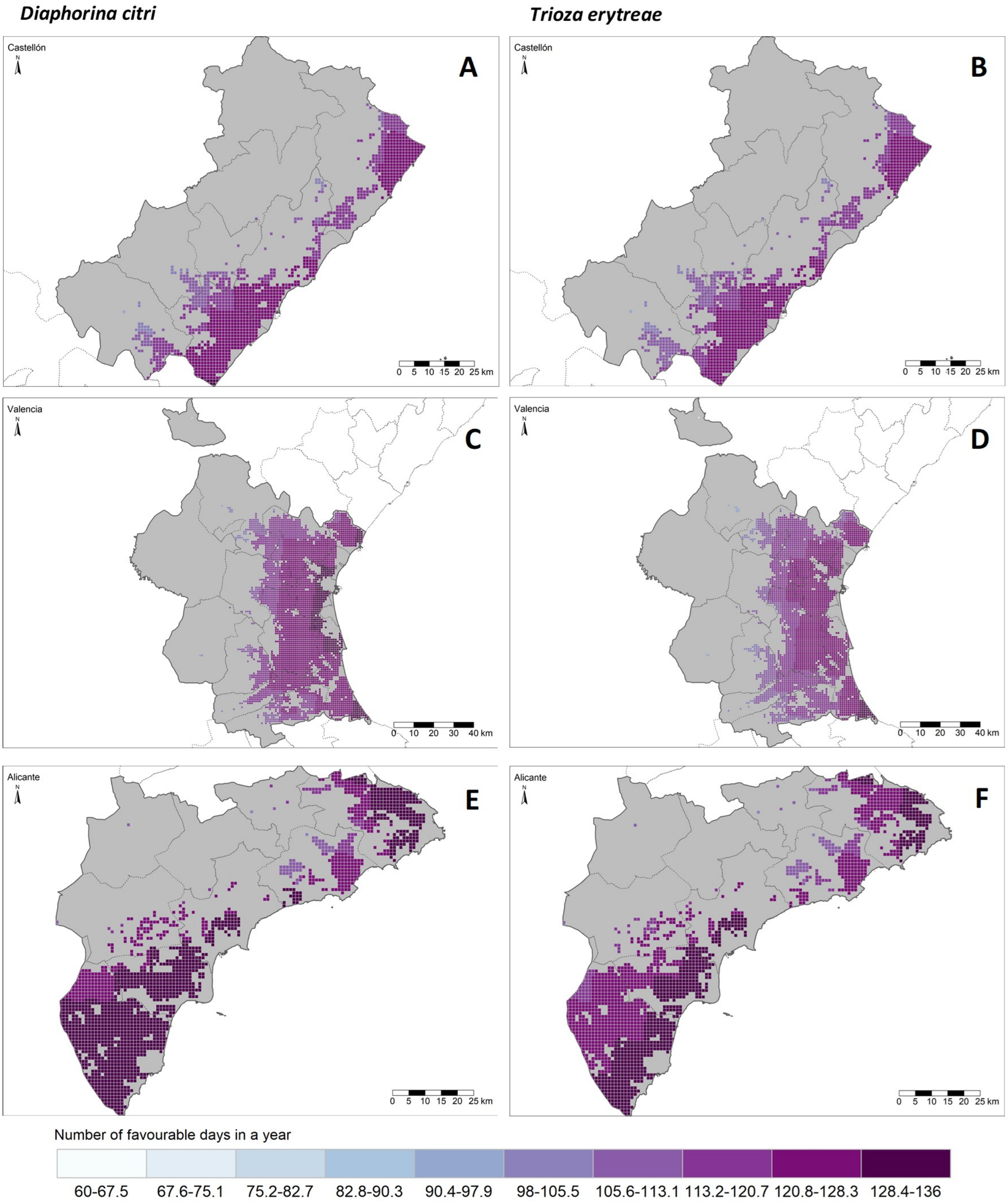

Figure SA2. Number of favorable days per year estimated for *Diaphorina citri* (A,C,E) and *Trioza erytreae* (B,D,F) disaggregated at province level (NUTS 3): Castellón (A,B), Valencia (C,D) and Alicante (E,F).

ACKNOWLEDGMENTS

We thank César Monzó (IVIA), Aranzazu Moreno and Alberto Fereres (IAS-CSIC), Amílcar Duarte, Tomás Magalhães and Rita Poeria (Univ. Algarve), Jacinto Benhadi (Inst. P. Bragança), Silvio Lopes

(Fundecitrus) and Alessandra Gentile (Univ. Catania) for their time, their knowledge and for a great deal of helpful, well-documented information about *D.citri* and *T. erytreae*. We would also like to thank all of them for their useful comments about the possible climatic requirements of the vectors in our study area.

REFERENCES

Aidoo, O. F., Tanga, C. M., Azrag, A. G., Mohamed, S. A., Khamis, F. M., Rasowo, B. A., et al. (2022). Temperature-based phenology model of African citrus triozid (*Trioza erytreae* Del Guercio): Vector of citrus greening disease. *Journal of Applied Entomology* 146, 88–97

Annecke, D. and Cilliers, C. (1963). The citrus psylla, *Trioza erytreae* (Del Guercio), and its parasites. *South African Journal of Agricultural Science* 6, 187–191

Boina, D. R., Meyer, W. L., Onagbola, E. O., and Stelinski, L. L. (2009). Quantifying dispersal of *Diaphorina citri* (Hemiptera: Psyllidae) by immunomarking and potential impact of unmanaged groves on commercial citrus management. *Environmental entomology* 38, 1250–1258

Bouvet, J. P. R., Urbaneja, A., and Monzó, C. (2019). Effects of citrus overwintering predators, host plant phenology and environmental variables on aphid infestation dynamics in clementine citrus. *Journal of Economic Entomology* 112, 1587–1597

Bové, J. (2006). Huanglongbing: a destructive, newly-emerging, century-old disease of citrus. *Journal of plant pathology* , 7–37

Catling, H. (1969a). The bionomics of the South African citrus psylla, *Trioza erytreae* (Del Guercio) (Homoptera: Psyllidae) I. The influence of the flushing rhythm of citrus and factors which regulate flushing. *Journal of the Entomological Society of Southern Africa* 32, 191–208

Catling, H. (1969b). The bionomics of the South African citrus psylla, *Trioza erytreae* (Del Guercio) (Homoptera: Psyllidae) III. The influence of extremes of weather on survival. *Journal of the Entomological Society of Southern Africa* 32, 273–290

Catling, H. (1972). The bionomics of the South African citrus psylla, *Trioza erytreae* (Del Guercio) (Homoptera: Psyllidae) VI. Final population studies and a discussion of population dynamics. *Journal of the Entomological Society of Southern Africa* 35, 235–251

Catling, H. and Green, G. (1972). The influence of weather on the survival and population fluctuations of *Trioza erytreae* (́Del Guercio)—a vector of greening. In *International Organization of Citrus Virologists Conference Proceedings (1957-2010)*. vol. 5

Catling, H. et al. (1970). Distribution of the psyllid vectors of citrus greening disease, with notes on the biology and bionomics of *Diaphorina citri*. *FAO Plant Protection Bulletin* 18, 8–15

Catling, H. D. (1973). Notes on the biology of the South African citrus psylla, *Trioza erytreae* (Del Guercio) (Homoptera: Psyllidae). *Journal of the Entomological Society of Southern Africa* , 299–306

Chiyaka, C., Singer, B. H., Halbert, S. E., Morris Jr, J. G., and van Bruggen, A. H. (2012). Modeling huanglongbing transmission within a citrus tree. *Proceedings of the National Academy of Sciences* 109, 12213–12218

Cifuentes-Arenas, J. C., de Goes, A., de Miranda, M. P., Beattie, G. A. C., and Lopes, S. A. (2018). Citrus flush shoot ontogeny modulates biotic potential of *Diaphorina citri*. *PLoS One* 13, e0190563

Cocuzza, G., Urbaneja, A., Hernández-Suárez, E., Siverio, F., Di Silvestro, S., Tena, A., et al. (2017). A review on *Trioza erytreae* (African citrus psyllid), now in mainland Europe, and its potential risk as vector of huanglongbing (hlb) in citrus. *Journal of Pest Science* , 1–17doi:ttps://doi.org/10.1007/s10340-016-0804-1

Croxton, S. D. and Stansly, P. A. (2014). Metalized polyethylene mulch to repel Asian citrus psyllid, slow spread of huanglongbing and improve growth of new citrus plantings. *Pest Management Science* 70, 318–323

Garcia-Marí, F., Granda, C., Zaragoza, S., and Agusti, M. (2002). Impact of *Phyllocnistis citrella* (Lepidoptera: Gracillariidae) on leaf area development and yield of mature citrus trees in the mediterranean area. *Journal of Economic Entomology* 95, 966–974

Grafton-Cardwell, E. E., Stelinski, L. L., and Stansly, P. A. (2013). Biology and management of Asian citrus psyllid, vector of the huanglongbing pathogens. *Annual Review of Entomology* 58, 413–432

Green, G. e. and Catling, H. (1971). Weather-induced mortality of the citrus psylla, *Trioza erytreae* (Del Guercio)(Homoptera: Psyllidae), a vector of greening virus, in some citrus producing areas of southern Africa. *Agricultural Meteorology* 8, 305–317

Halbert, S. E. and Manjunath, K. L. (2004). Asian citrus psyllids (Sternorrhyncha: Psyllidae) and greening disease of citrus: a literature review and assessment of risk in Florida. *Florida entomologist* 87, 330–353

Halbert, S. E., Manjunath, K. L., Ramadugu, C., Brodie, M. W., Webb, S. E., and Lee, R. F. (2010). Trailers transporting oranges to processing plants move Asian citrus psyllids. *Florida Entomologist* 93, 33–38

Hall, D. G. (2020). Asian citrus psyllid life cycle and developmental biology. In *Asian citrus psyllid: biology, ecology and management of the Huanglongbing vector* (CABI Wallingford UK). 1–11

Hall, D. G. and Gottwald, T. R. (2011). Pest management practices aimed at curtailing citrus huanglongbing disease. *Outlooks on Pest Management* 22, 189

Hermoso De Mendoza, A., Esteve, R., Llorens, J., Michelena, J. M., et al. (2012). Evolución global y por colonias de los pulgones (Hemiptera, Aphididae) y sus enemigos naturales en clementinos y limoneros valencianos. *Boletín de sanidad vegetal. Plagas.* 38, 61–72

Hodkinson, I. D. (2009). Life cycle variation and adaptation in jumping plant lice (Insecta: Hemiptera: Psylloidea): a global synthesis. *Journal of Natural History* 43, 65–179

Honěk, A., Kocourek, F., et al. (1990). Temperature and development time in insects: a general relationship between thermal constants. *Zoologische Jahrbücher, Abteilung für Systematik, Ökologie und Geographie der Tiere* 117, 401–439

Jenkins, D. A., Hall, D. G., and Goenaga, R. (2015). *Diaphorina citri* (Hemiptera: Liviidae) abundance in Puerto Rico declines with elevation. *Journal of Economic Entomology* 108, 252–258

Lebbal, S. and Laamari, M. (2016). Population dynamics of aphids (Aphididae) on orange (*Citrus sinensis* 'thomson navel') and mandarin (*Citrus reticulata* 'blanco'). *Acta Agriculturae Slovenica* 107, 137–145

Lewis-Rosenblum, H., Martini, X., Tiwari, S., and Stelinski, L. L. (2015). Seasonal movement patterns and long-range dispersal of Asian citrus psyllid in Florida citrus. *Journal of Economic Entomology* 108, 3–10

Liu, Y. and Tsai, J. (2000). Effects of temperature on biology and life table parameters of the Asian citrus psyllid, *Diaphorina citri* Kuwayama (Hemiptera: Psyllidae). *Annals of Applied Biology* , 201–206

Martini, X., Pelz-Stelinski, K. S., and Stelinski, L. L. (2016). Factors affecting the overwintering abundance of the Asian citrus psyllid (Hemiptera: Liviidae) in Florida citrus (Sapindales: Rutaceae) orchards. *Florida Entomologist* 99, 178–186

Martini, X. and Stelinski, L. L. (2017). Influence of abiotic factors on flight initiation by Asian citrus psyllid (Hemiptera: Liviidae). *Environmental Entomology* 46, 369–375

Monzó, C. and Vanaclocha, P. (2023). *Diaphorina citri* (Asian citrus psyllid). *CABI Compendium. CABI International*

Moran, V. e. and Buchan, P. (1975). Oviposition by the citrus psylla, *Trioza erytreae* (Homoptera: Psyllidae), in relation to leaf hardness. *Entomologia experimentalis et applicata* 18, 96–104

Samways, M. and Manicom, B. (1983). Immigration, frequency distributions and dispersion patterns of the psyllid *Trioza erytreae* (Del Guercio) in a citrus orchard. *Journal of Applied Ecology* , 463–472

Sétamou, M. and Bartels, D. W. (2015). Living on the edges: spatial niche occupation of Asian citrus psyllid, *Diaphorina citri* Kuwayama (Hemiptera: Liviidae), in citrus groves. *PloS one* 10, e0131917

Sétamou, M., Flores, D., Victor French, J., and Hall, D. G. (2008). Dispersion patterns and sampling plans for *Diaphorina citri* (Hemiptera: Psyllidae) in citrus. *Journal of Economic Entomology* 101, 1478–1487

Tamesse, J. L. and Messi, J. (2004). Facteurs influençant la dynamique des populations du psylle africain des agrumes *Trioza erytreae* Del Guercio (Hemiptera: Triozidae) au Cameroun. *International Journal of Tropical Insect Science* 24, 213–227

Tiwari, S., Lewis-Rosenblum, H., Pelz-Stelinski, K., and Stelinski, L. L. (2010). Incidence of *Candidatus* Liberibacter asiaticus infection in abandoned citrus occurring in proximity to commercially managed groves. *Journal of economic entomology* 103, 1972–1978

Tomaseto, A., Miranda, M., Moral, R., De Lara, I., Fereres, A., and Lopes, J. R. S. (2018). Environmental conditions for *Diaphorina citri* Kuwayama (Hemiptera: Liviidae) take-off. *Journal of applied entomology* 142, 104–113

Udell, B., Monzó, C., Paris, T., Allan, S., and Stansly, P. A. (2017). Influence of limiting and regulating factors on populations of Asian citrus psyllid and the risk of insect and disease outbreaks. *Annals of Applied Biology* 171, 70–88

van den Berg, M. and Deacon, V. (1988). Dispersal of the citrus psylla, *Trioza erytreae* (Hemiptera: Triozidae), in the absence of its host plants. *Phytophylactica* 20, 361–368
