# Supplementary material for: Risk-based regionalization approach for area-wide management of HLB vectors in the Mediterranean Basin
Source: Front Plant Sci. 2023 Dec 4;14:1256935. doi: 10.3389/fpls.2023.1256935 (PMC10725980; doi:10.3389/fpls.2023.1256935)
Supplement: Supplementary Material B — Pest Management Areas (PMAs) descriptive. [file DataSheet_2.pdf]

Supplementary Material B. Pest Management Areas (PMAs) descriptive

1 SUPPLEMENTARY FIGURES

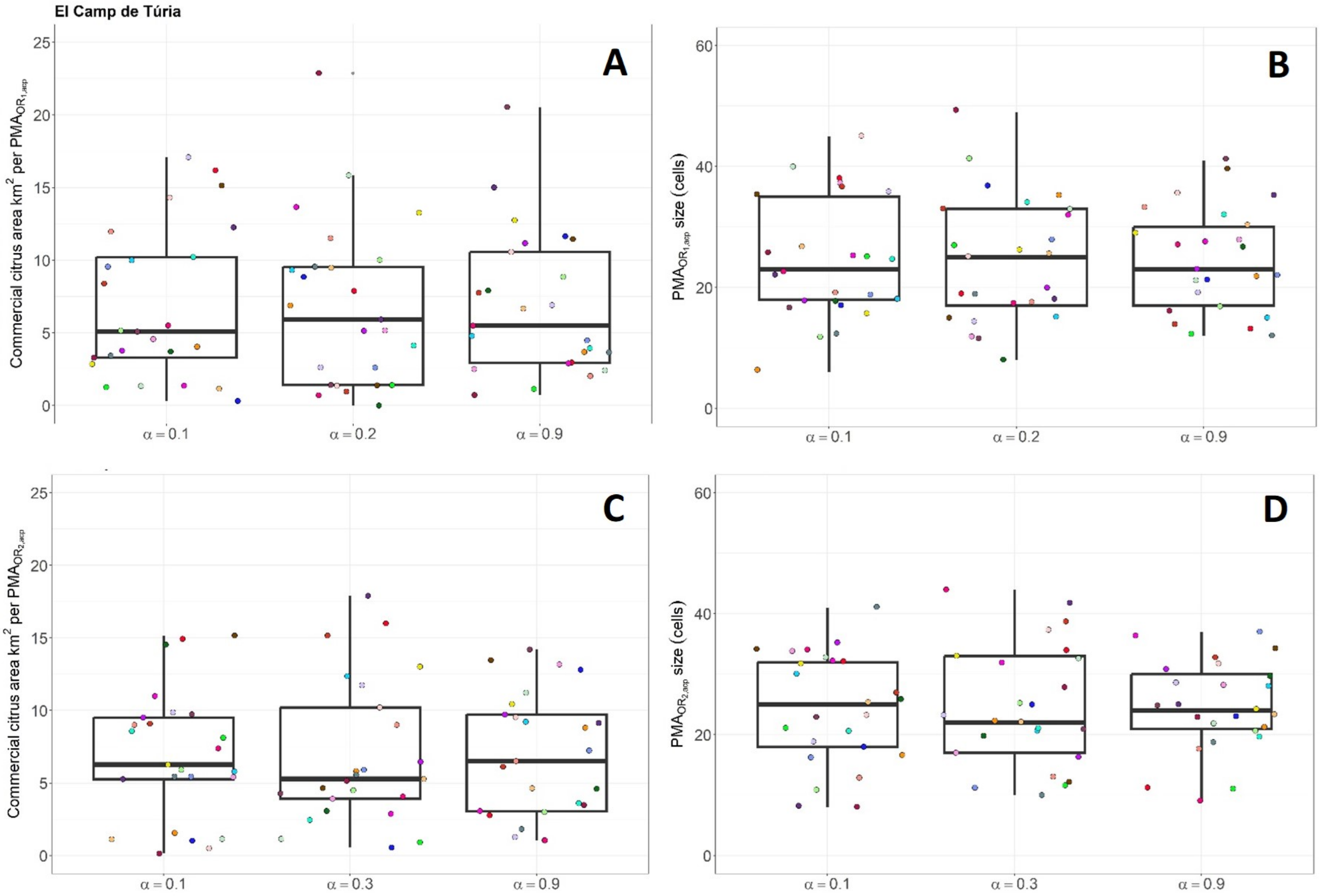

Figure SB1: Individual and overall distribution of the commercial citrus area (km^2) and number of cells in PMAs$_{OR_{1,acp}}$ (A,B) and PMAs$_{OR_{2,acp}}$ (C,D) in "El Camp de Túria" county. Box-and-whisker plots for A–D: central line represents the median value; box limits represents the first (Q_1) and third (Q_3) quantiles; upper whisker represents $min(max(x), Q_3 + 1.5IQR)$; lower whisker represents $max(min(x), Q_1 - 1.5IQR)$; $IQR = Q_3 - Q_1$; outliers are represented by dots.

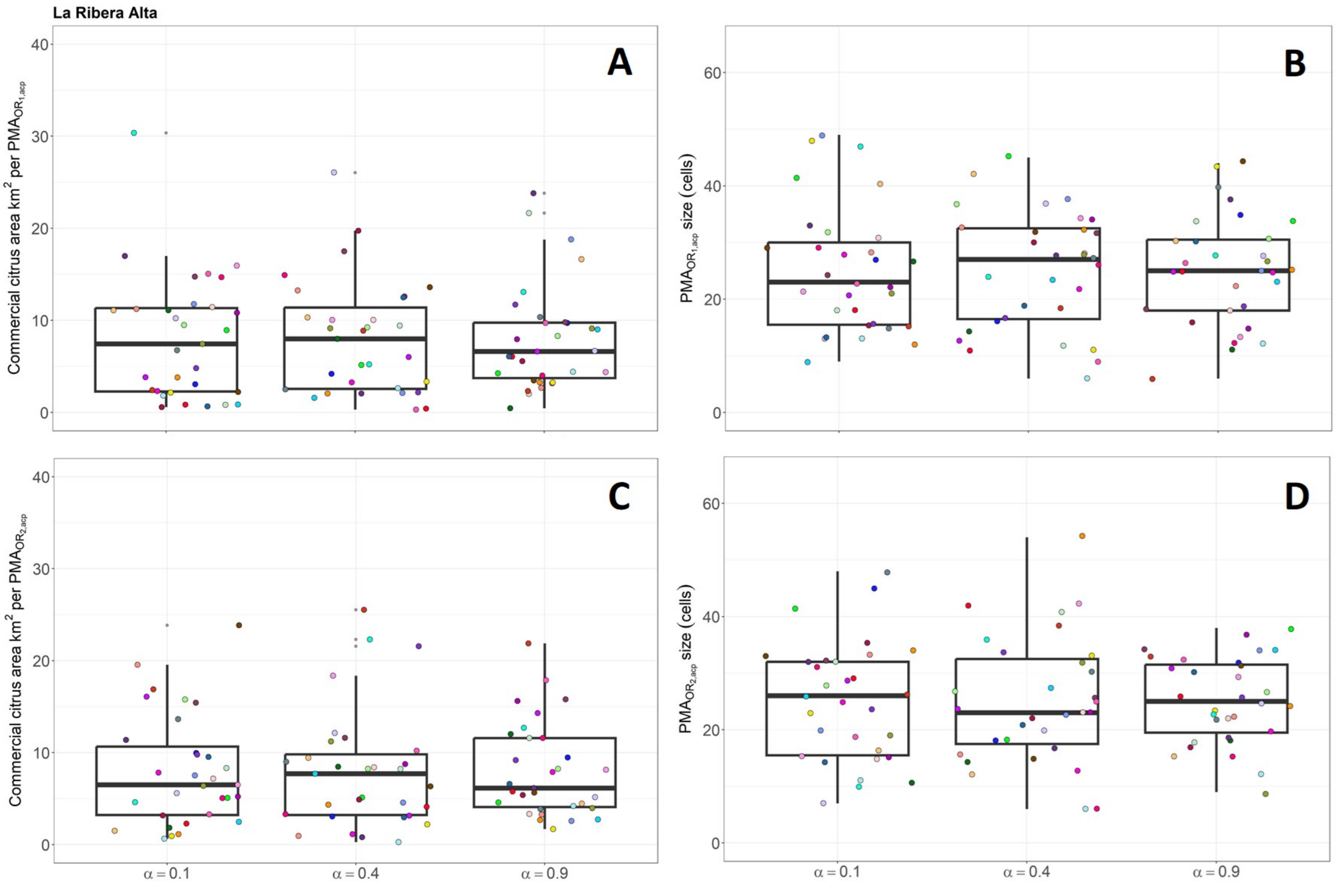

Figure SB2: Individual and overall distribution of the commercial citrus area (km^2) and number of cells in PMAs$_{OR_{1,acp}}$ (A,B) and PMAs$_{OR_{2,acp}}$ (C,D) in "La Ribera Alta" county. Box-and-whisker plots for A–D: central line represents the median value; box limits represents the first (Q_1) and third (Q_3) quantiles; upper whisker represents $min(max(x), Q_3 + 1.5IQR)$; lower whisker represents $max(min(x), Q_1 - 1.5IQR)$; $IQR = Q_3 - Q_1$; outliers are represented by dots.

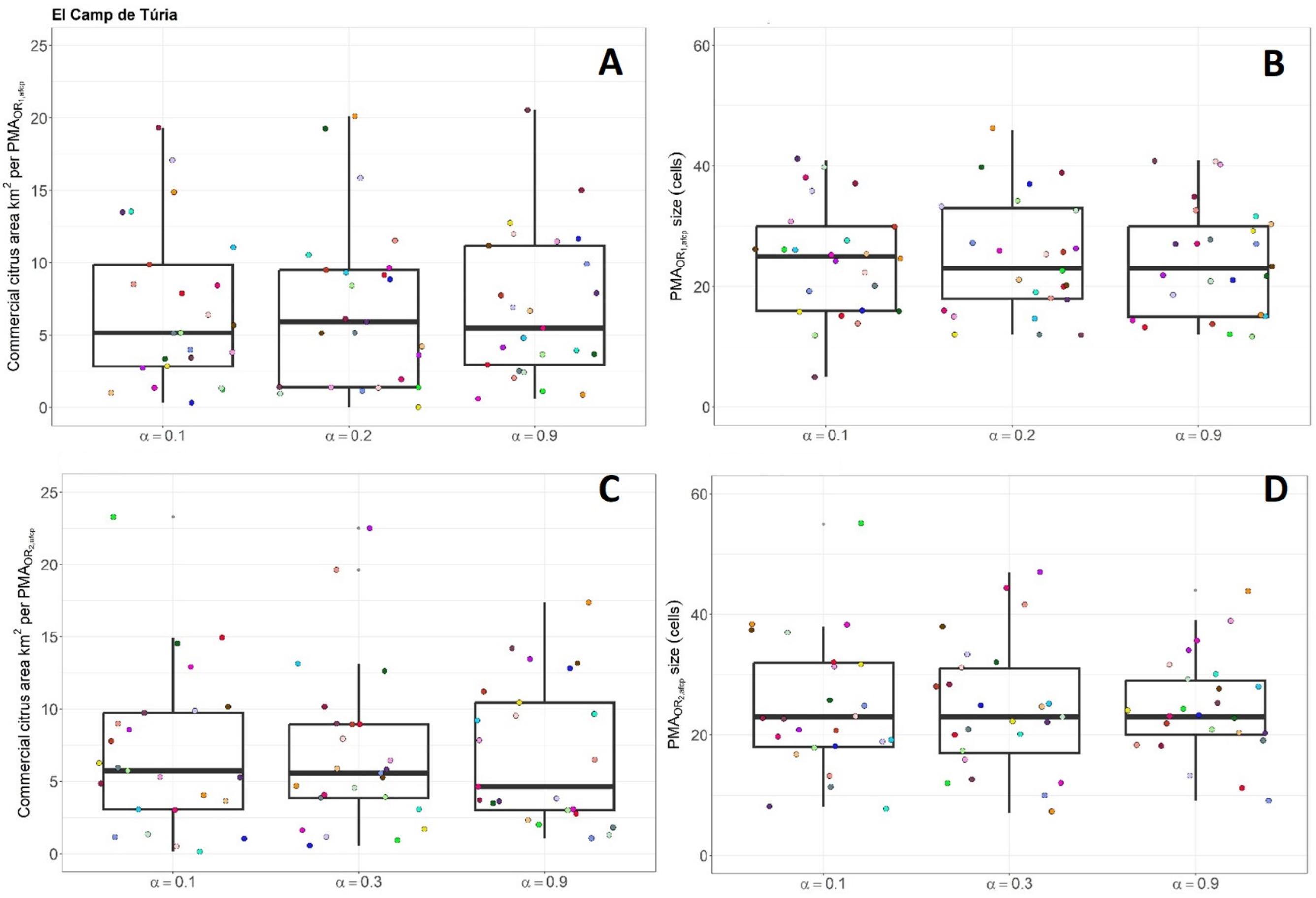

Figure SB3: Individual and overall distribution of the commercial citrus area (km^2) and number of cells in PMAs$_{OR_{1,afcp}}$ (A,B) and PMAs$_{OR_{2,afcp}}$ (C,D) in "El Camp de Túria" county. Box-and-whisker plots for A–D: central line represents the median value; box limits represents the first (Q_1) and third (Q_3) quantiles; upper whisker represents $min(max(x), Q_3 + 1.5IQR)$; lower whisker represents $max(min(x), Q_1 - 1.5IQR)$; $IQR = Q_3 - Q_1$; outliers are represented by dots.

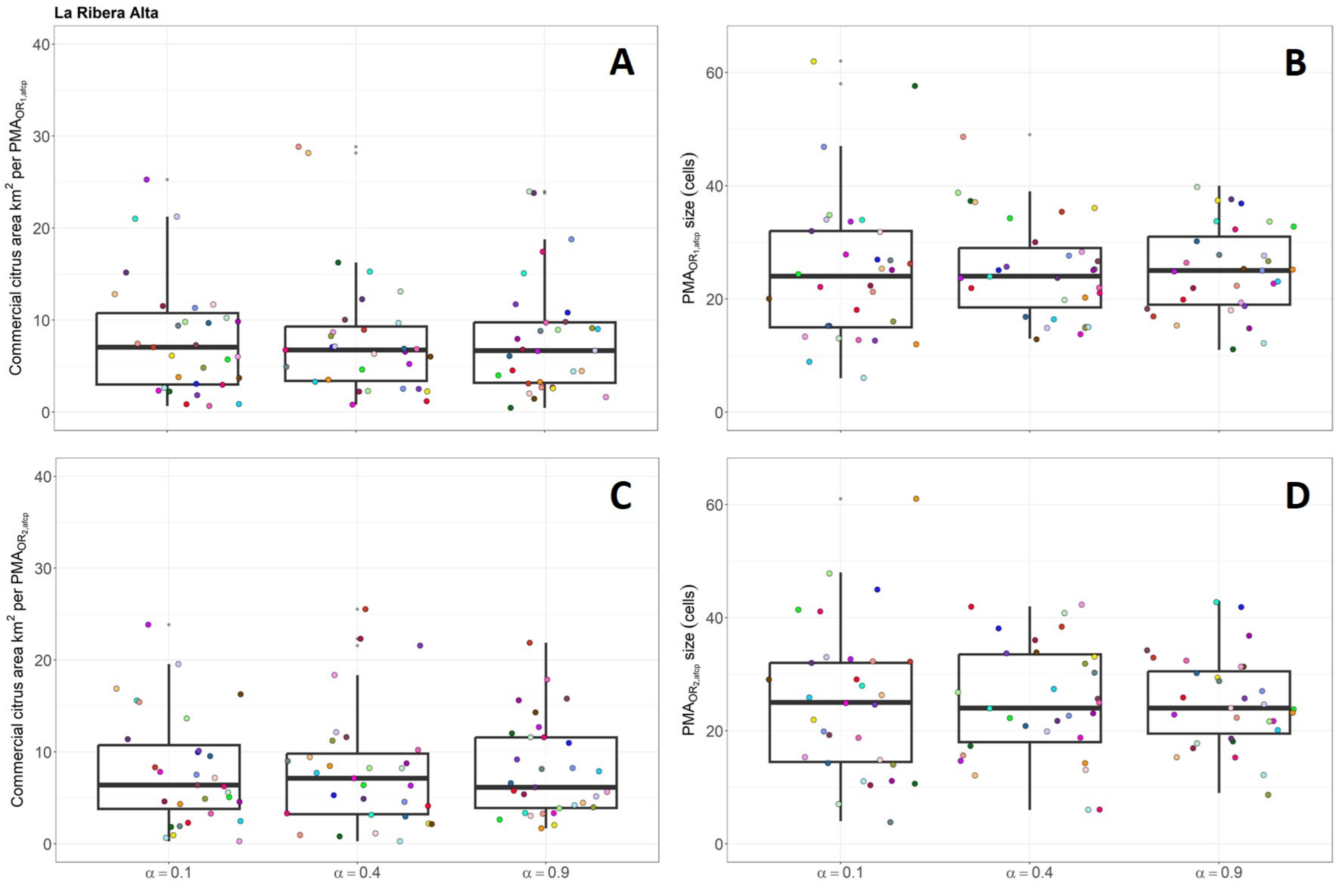

Figure SB4: Individual and overall distribution of the commercial citrus area (km^2) and number of cells in PMAs$_{OR_{1,afcp}}$ (A,B) and PMAs$_{OR_{2,afcp}}$ (C,D) in "La Ribera Alta" county. Box-and-whisker plots for A–D: central line represents the median value; box limits represents the first (Q_1) and third (Q_3) quantiles; upper whisker represents $min(max(x), Q_3 + 1.5IQR)$; lower whisker represents $max(min(x), Q_1 - 1.5IQR)$; $IQR = Q_3 - Q_1$; outliers are represented by dots.

ACKNOWLEDGMENTS

We thank César Monzó (IVIA), Aranzazu Moreno and Alberto Fereres (IAS-CSIC), Amílcar Duarte, Tomás Magalhães and Rita Poeria (Univ. Algarve), Jacinto Benhadi (Inst. P. Bragança), Silvio Lopes (Fundecitrus) and Alessandra Gentile (Univ. Catania) for their time, their knowledge and for a great deal of helpful, well-documented information about *D. citri* and *T. erytreae*. We would also like to thank all of them for their useful comments about the possible climatic requirements of the vectors in our study area.
