# Supplementary material for: Risk-based regionalization approach for area-wide management of HLB vectors in the Mediterranean Basin
Source: Front Plant Sci. 2023 Dec 4;14:1256935. doi: 10.3389/fpls.2023.1256935 (PMC10725980; doi:10.3389/fpls.2023.1256935)
Supplement: Supplementary file 3 [file DataSheet_3.pdf]

# Readme. Data and code description

## 1 CODE DESCRIPTION

Source code (`R` language) for manuscript "Risk-based regionalization approach for area-wide management of HLB vectors in the Mediterranean Basin"

For questions, comments or remarks about the code please contact E. Lázaro (`lazaro_ele@gva.es`)

Source code has been structured in three main folders:

- **`data`**, which contains both the spatial objects and the databases necessary to reproduce all the methodological approaches considered in the manuscript.
- **`code`**, which contains the scripts to address i) both the estimation of risk factors and overall risks; ii) the implementation of the regionalization algorithm to estimate the size and location of the pest management areas (PMAs); and iii) the implementation of the sensitivity analysis to assess the performance of the regionalisation algorithm.
- **`results`**. An auxiliary folder where the graphic and database files to support/analyse the results will be stored.

**Note:** To run the code it is provided a clip of the study area which corresponds to "La Ribera Alta" county.

### 1.1 Data

**Subfolder contents**:

- **boundaries**. This folder contains the .shp. file and the auxiliary files of the boundaries of "La Ribera Alta" county.
- **citrus_density**. This folder contains the .shp file and the auxiliary files which specifies at 1 km$^2$ cell level two cell identifiers (`grd_id1`, `grd_id2` ), the commercial citrus area (`com`) and its division under conventional (`con`), organic (`org`) and abandoned (`abn`) management as well as the citrus residential area (`res`). Areas are in km$^2$.
- **climatic**. This folder contains 4 .RData files:
  - `df_tmax09_18_ra.RData` with daily maximum temperatures 2009-2018 from 15 February to 30 April, June and October*.
  - `df_tmin09_18_ra.RData` with daily minimum temperatures 2009-2018 from 15 February to 30 April, June and October*.
  - `df_tmean09_18_ra.RData` with daily mean temperatures from 2009-2018 from 15 February to 30 April, June and October*.

- `df_rhmin09_18_ra.RData` with daily minimum relative humidities 2009-2018 from 15 February to 30 April, June and October*.

  *. It is assumed that under Mediterranean conditions: i) from 15 February to 30 April, ii) June and iii) October, are the three major leaf flushing periods.

- **corridors**. This folder contains the .shp. file and the auxiliary files of main transport corridors within "La Ribera Alta" county.
- **citrus_risks**. This folder contains the .shp file and the auxiliary files which specifies at 1 km$^2$ cell level two cell identifiers (`grd_id1`, `grd_id2` ), the estimates of the individual risk factors (`r_png_dc`, `r_nfd_dc`, `r_png_te`, `r_nfd_te`,`r_com`, `r_abn`,`r_org`, `r_res`, `r_tra`) and the overall risks (`or1_dc`, `or2_dc`, `or1_te`, `or2_te`). The proposed nomenclature for naming risk factors and overall risks is aligned with that defined in the manuscript. This .shp file will be assembled after the execution of the `1_risk_estimates.R`

## 1.2 Code

**Scripts**:

- `1_risk_estimates.R`. Script containing the implementation for estimating the individual risk factors and the overall risks.

  **sessionInfo():**

```
R version 4.2.0 (2022-04-22 ucrt)
Platform: x86_64-w64-mingw32/x64 (64-bit)
Running under: Windows 10 x64 (build 19045)

Matrix products: default

locale:
[1] LC_COLLATE=Spanish_Spain.utf8  LC_CTYPE=Spanish_Spain.utf8
[3] LC_MONETARY=Spanish_Spain.utf8 LC_NUMERIC=C
[5] LC_TIME=Spanish_Spain.utf8

attached base packages:
[1] stats     graphics  grDevices utils     datasets  methods
[7] base

other attached packages:
[1] scales_1.2.1 sf_1.0-7

loaded via a namespace (and not attached):
 [1] Rcpp_1.0.8.3       rstudioapi_0.13    magrittr_2.0.3
 [4] units_0.8-0        munsell_0.5.0      tidyselect_1.1.2
 [7] colorspace_2.0-3   R6_2.5.1           rlang_1.0.6
[10] fansi_1.0.3        dplyr_1.0.9        tools_4.2.0
[13] grid_4.2.0         KernSmooth_2.23-20 utf8_1.2.2
```

```
[16] cli_3.6.0          e1071_1.7-9        DBI_1.1.2
[19] ellipsis_0.3.2     class_7.3-20       assertthat_0.2.1
[22] tibble_3.1.7       lifecycle_1.0.3    purrr_0.3.4
[25] vctrs_0.5.2        glue_1.6.2         proxy_0.4-26
[28] compiler_4.2.0     pillar_1.8.1       generics_0.1.2
[31] classInt_0.4-3     pkgconfig_2.0.3
```

- `2_pmas_algorithm.R`. Script containing the implementation of the algorithm to estimate size and location of the PMAs. After the execution of this script, the following files will be stored in the results folder: i) `cr.RData`, which contains numerical information about the proportion of explained pseudo-inertia and the normalised proportion of explained pseudo-inertia for different $\alpha$ values (i.e., from $\alpha = 0$ to $\alpha = 1$) by 0.1; ii) `alpha.RData`, which contains the selected values of $\alpha$; and iii) `pmas_list.RData`, a list of 3 elements, each one for one of the three $\alpha$ values considered. Each element contains at 1 km$^2$ cell level an assigned number that identifies its corresponding PMA.

  **sessionInfo():**

```
R version 4.2.0 (2022-04-22 ucrt)
Platform: x86_64-w64-mingw32/x64 (64-bit)
Running under: Windows 10 x64 (build 19045)

Matrix products: default

locale:
[1] LC_COLLATE=Spanish_Spain.utf8  LC_CTYPE=Spanish_Spain.utf8
[3] LC_MONETARY=Spanish_Spain.utf8 LC_NUMERIC=C
[5] LC_TIME=Spanish_Spain.utf8

attached base packages:
[1] stats     graphics  grDevices utils     datasets  methods
[7] base

other attached packages:
[1] sp_1.4-7     ClustGeo_2.1 sf_1.0-7

loaded via a namespace (and not attached):
 [1] Rcpp_1.0.8.3       rstudioapi_0.13    magrittr_2.0.3
 [4] units_0.8-0        tidyselect_1.1.2   lattice_0.20-45
 [7] R6_2.5.1           rlang_1.0.6        fansi_1.0.3
[10] dplyr_1.0.9        tools_4.2.0        grid_4.2.0
[13] KernSmooth_2.23-20 utf8_1.2.2         cli_3.6.0
[16] e1071_1.7-9        DBI_1.1.2          ellipsis_0.3.2
[19] class_7.3-20       assertthat_0.2.1   tibble_3.1.7
[22] lifecycle_1.0.3    purrr_0.3.4        vctrs_0.5.2
[25] glue_1.6.2         proxy_0.4-26       compiler_4.2.0
[28] pillar_1.8.1       generics_0.1.2     classInt_0.4-3
[31] pkgconfig_2.0.3
```

- `3_pmas_results.R`. Script containing the implementation of the sensitivity analysis carried out to assess the performance of the regionalization algorithm under the three explored configurations

defined according to the $\alpha$ parameter value. After the execution of this script, a detailed graphical and numerical description of the estimated PMAs is obtained under the different $\alpha$ configurations evaluated. Additionally, a graphical and numerical description of the sensitivity analysis proposed in the manuscript is also covered. Graphical and numerical objects are programmed to be stored in results folder.

**sessionInfo():**

```
R version 4.2.0 (2022-04-22 ucrt)
Platform: x86_64-w64-mingw32/x64 (64-bit)
Running under: Windows 10 x64 (build 19045)

Matrix products: default

locale:
[1] LC_COLLATE=Spanish_Spain.utf8  LC_CTYPE=Spanish_Spain.utf8
[3] LC_MONETARY=Spanish_Spain.utf8 LC_NUMERIC=C
[5] LC_TIME=Spanish_Spain.utf8

attached base packages:
[1] stats     graphics  grDevices utils     datasets  methods
[7] base

other attached packages:
[1] clv_0.3-2.2      class_7.3-20     cluster_2.1.3
[4] ggplot2_3.4.0    tmap_3.3-3       Polychrome_1.5.1
[7] xtable_1.8-4     dplyr_1.0.9      sf_1.0-7

loaded via a namespace (and not attached):
 [1] tidyselect_1.1.2    terra_1.5-21        purrr_0.3.4
 [4] lattice_0.20-45     colorspace_2.0-3    vctrs_0.5.2
 [7] generics_0.1.2      htmltools_0.5.2     stars_0.5-5
[10] viridisLite_0.4.1   base64enc_0.1-3     utf8_1.2.2
[13] XML_3.99-0.9        rlang_1.0.6         e1071_1.7-9
[16] pillar_1.8.1        withr_2.5.0         glue_1.6.2
[19] DBI_1.1.2           sp_1.4-7            RColorBrewer_1.1-3
[22] lifecycle_1.0.3     munsell_0.5.0       gtable_0.3.1
[25] raster_3.5-15       htmlwidgets_1.5.4   leafsync_0.1.0
[28] codetools_0.2-18    fastmap_1.1.0       crosstalk_1.2.0
[31] parallel_4.2.0      fansi_1.0.3         leafem_0.2.0
[34] Rcpp_1.0.8.3        KernSmooth_2.23-20  scales_1.2.1
[37] classInt_0.4-3      lwgeom_0.2-8        scatterplot3d_0.3-42
[40] leaflet_2.1.1       abind_1.4-5         png_0.1-7
[43] digest_0.6.29       tmaptools_3.1-1     grid_4.2.0
[46] cli_3.6.0           tools_4.2.0         magrittr_2.0.3
[49] proxy_0.4-26        tibble_3.1.7        dichromat_2.0-0.1
[52] pkgconfig_2.0.3     ellipsis_0.3.2      assertthat_0.2.1
[55] rstudioapi_0.13     R6_2.5.1            units_0.8-0
[58] compiler_4.2.0
```

## Comments

- The scripts have been created under UTF-8 encoding.
- To run the scripts it is provided a clip of the study area which corresponds to “La Ribera Alta” county.
- The scripts have been executed with the `R` version 4.2.0 (2022-04-22 ucrt); Platform: x86_64-w64-mingw32/x64 (64-bit); Running under: Windows 10 x64 (build 19045)
- To execute the code, `R` scripts should be run in the order specified in the file name (i.e., 1_, 2_, ...)
